# Global Patterns in Ecological Indicators of Marine Food Webs: A Modelling Approach

**DOI:** 10.1371/journal.pone.0095845

**Published:** 2014-04-24

**Authors:** Johanna Jacomina Heymans, Marta Coll, Simone Libralato, Lyne Morissette, Villy Christensen

**Affiliations:** 1 Scottish Association for Marine Science, Scottish Marine Institute, Oban, Argyll, United Kingdom; 2 Institute of Marine Science (ICM-CSIC), Passeig Marítim de la Barceloneta, Barcelona, Spain; 3 Ecopath International Initiative Research Association, Barcelona, Spain; 4 Department of Oceanography, OGS (Istituto Nazionale di Oceanografia e di Geofisica Sperimentale), Borgo Grotta Gigante, Sgonico, Zgonik (TS), Italy; 5 M – Expertise Marine, Sainte-Luce, Quebec, Canada; 6 Fisheries Centre, University of British Columbia, Vancouver, Canada; Dauphin Island Sea Lab, United States of America

## Abstract

**Background:**

Ecological attributes estimated from food web models have the potential to be indicators of good environmental status given their capabilities to describe redundancy, food web changes, and sensitivity to fishing. They can be used as a baseline to show how they might be modified in the future with human impacts such as climate change, acidification, eutrophication, or overfishing.

**Methodology:**

In this study ecological network analysis indicators of 105 marine food web models were tested for variation with traits such as ecosystem type, latitude, ocean basin, depth, size, time period, and exploitation state, whilst also considering structural properties of the models such as number of linkages, number of living functional groups or total number of functional groups as covariate factors.

**Principal findings:**

Eight indicators were robust to model construction: relative ascendency; relative overhead; redundancy; total systems throughput (TST); primary production/TST; consumption/TST; export/TST; and total biomass of the community. Large-scale differences were seen in the ecosystems of the Atlantic and Pacific Oceans, with the Western Atlantic being more complex with an increased ability to mitigate impacts, while the Eastern Atlantic showed lower internal complexity. In addition, the Eastern Pacific was less organised than the Eastern Atlantic although both of these systems had increased primary production as eastern boundary current systems. Differences by ecosystem type highlighted coral reefs as having the largest energy flow and total biomass per unit of surface, while lagoons, estuaries, and bays had lower transfer efficiencies and higher recycling. These differences prevailed over time, although some traits changed with fishing intensity. Keystone groups were mainly higher trophic level species with mostly top-down effects, while structural/dominant groups were mainly lower trophic level groups (benthic primary producers such as seagrass and macroalgae, and invertebrates). Keystone groups were prevalent in estuarine or small/shallow systems, and in systems with reduced fishing pressure. Changes to the abundance of key functional groups might have significant implications for the functioning of ecosystems and should be avoided through management.

**Conclusion/significance:**

Our results provide additional understanding of patterns of structural and functional indicators in different ecosystems. Ecosystem traits such as type, size, depth, and location need to be accounted for when setting reference levels as these affect absolute values of ecological indicators. Therefore, establishing absolute reference values for ecosystem indicators may not be suitable to the ecosystem-based, precautionary approach. Reference levels for ecosystem indicators should be developed for individual ecosystems or ecosystems with the same typologies (similar location, ecosystem type, etc.) and not benchmarked against all other ecosystems.

## Introduction

Natural resource management approaches have been under development for decades, driven by an increasing need to understand the effect of anthropogenic impacts on ecosystems [1], [2]. Often, it was assumed that management could be based on population dynamics at the individual species population level [3]. However, it is now clear that there are trade-offs in management [4]–[8] and that community effects therefore must be considered. An example of these trade-offs is food web effects due to competition or predation [2], [9], [10]. Ecosystems also have emergent properties that call for consideration of ecosystem structure and function in the management of marine resources [11]–[13].

Although detailed expert knowledge is essential for the management of an ecosystem, a general theory of ecosystem dynamics can help in defining aspects to be considered when conducting ecosystem based management. Such a theory would allow for extrapolation between systems, important given the lack of detailed information available on some systems. Food webs describe the interaction between species at different feeding levels and depict the flow of energy and matter in ecosystems. These predator-prey interactions are considered one of the main regulators of ecosystem dynamics [14], [15], and they partially mediate the way ecosystems respond to natural and human perturbations such as fishing, habitat degradation or environmental dynamics. Food web models are simplified representations of natural systems, which help us understand how biodiversity and ecosystems respond to changes. Creating food web models typically calls for quantitative modeling integrating food web dynamics and external factors such as environmental change or fishing.

The study of marine food webs and ecosystems faces difficulties with data collection and quantification of interactions, and the added difficulty of modeling ecosystem processes and dynamics [12]. Therefore, structural and functional properties of marine ecosystems are less known than their terrestrial and freshwater counterparts [12], [16]. Moreover, the quantification of many food web properties depends upon the modeling strategy and model structure as they co-vary with model components and links [12], [17], [18]. However, this lack of knowledge is changing. Ecological modelling applications have increased exponentially and a large body of standardized food web models have been constructed over the last three decades to quantitatively describe marine systems. The quantitative description of food web attributes is essential to advance our understanding of (i) ecosystem structure and functioning; and (ii) how to use ecological indicators to inform policy makers and managers [19]–[21].

The most widely applied ecosystem modelling approach is Ecopath with Ecosim (EwE, www.ecopath.org) [22]–[25], developed by Polovina [25] and adapted by Christensen and Pauly [26] and Walters [23], [27] into a comprehensive modelling tool [28]. EwE is currently composed of a core mass balance food web module (Ecopath), from which temporal (Ecosim) and spatial (Ecospace) dynamic simulations can be developed [24], [29]. EwE models represent complex food webs with non-linear and non-randomly distributed interactions, where each node or functional group of the web may be a species, a group of species, an ontogenetic stage of a species, or a detritus group [24]. Ecopath models are different from cascade models [30] and niche models [31], which are very useful to describe several food web structural properties. Niche and cascade models have been compared to results with EwE [32], but they do not account for the strength of ecological interactions.

**Figure 1:**
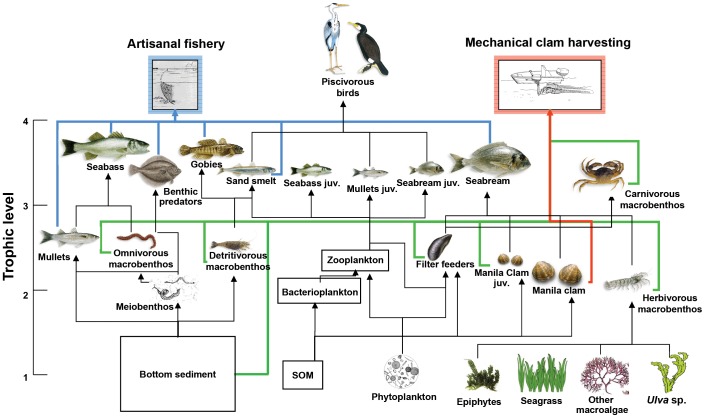
Food web diagram of the Venice lagoon with 27 nodes or funtional groups. Colors of flows depict different fishing target (artisanal fisheries in blue, and clam fishery in red) and non-target species (for clam harvesting, in green). Modified from Pranovi et al. [102].

The functional groups of EwE models are characterised by specific features such as abundance, biomass, and production, which provide means of measuring biological diversity. The nodes are linked by the strength of trophic (feeding) interactions, while Ecosim can represent non-feeding interactions such as mutualism or parasitism. Therefore, EwE models represent complex ecological networks with a series of properties that characterise food webs and are important for describing ecosystem structure and functioning and for evaluating conservation needs (Fig. 1 shows an illustrated example of a marine food web). Since food web models typically include all trophic levels from the lowest (i.e., primary producers and detritus) to highest (e.g., humans and apex predators), they are able to capture both bottom-up and top-down forcing dynamics.

The structural and functional properties of food webs can be quantified using ecological network analysis [22]. Ecological network analysis stems from the study in 1942 by Lindeman [33]. Trophic interactions and linkages were further conceptualized in food webs of energy transfers by EP Odum [34]. In 1980, Ulanowicz [35] developed indicators of ecosystem development such as ascendency [36].

The currently available EwE food web models provide relevant information to progress the study of a general theory of marine food web traits and dynamics. Taking advantage of the significant number of published marine food web models found in the literature (Table S1), this study investigates whether there are general patterns in ecological traits of marine food webs. To take into account the fact that models vary due to different development strategies, structural properties of the models (such as the number of total, living and non-living, versus only living functional groups, and total number of trophic links, called factors in this study) are used to test the robustness of these patterns by means of covariance analysis.

**Figure 2:**
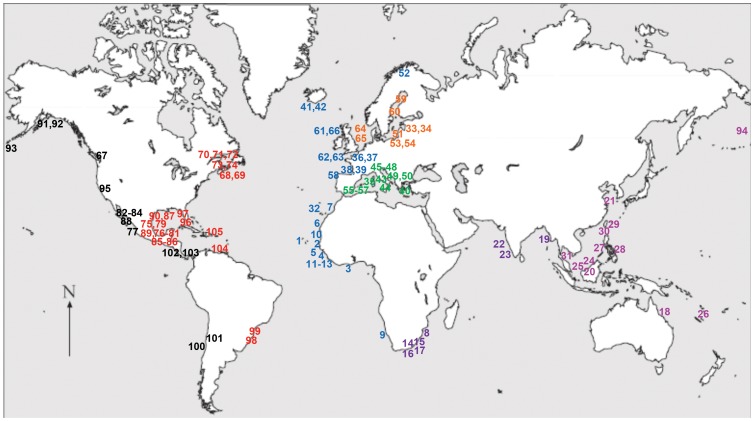
Distribution of food web model used in this study. Models are divided into Eastern Pacific (black), Western Atlantic (red), Eastern Atlantic (blue), North and Baltic Seas (orange), Mediterranean Sea (green), Indian Ocean (purple) and Eastern Pacific (pink). Each model is numbered on the graph according to its number in Table S1, where details and references to each model area are given.

This paper analyses 105 published EwE models distributed worldwide (Fig. 2) and their emergent ecological network analysis properties to characterise structural and functioning indicators of marine ecosystems. It also includes 26 indicators of ecosystem structure and function as defined in Table 1. The models represent a wide spatial diversity, covering ecosystems from coastal lagoons to the deep sea in all the world's oceans, and large temporal diversity, with ecosystems representing both past and recent timeframes (Fig. 2 & Table S1). To analyse the variation of general patterns in food web indicators, 7 traits were included in the analysis: (i) ecosystem type (coastal, shelf, slope, estuary, bay, lagoon or reef); (ii) latitude; (iii) ocean basin; (iv) depth; (v) size; (vi) period of time represented; and (vii) exploitation rate.

**Table 1:** Ecological and fisheries related indicators used in this comparison.

Acronym	Indicators	Units	Definition	Reference
**Ecological indicators**				
**TST**	Total System Throughput	t·km^−2^·y^−1^	The sum of all the flows through the ecosystem	[45]
**PP/TST**	Primary production/TST		Primary production over the sum of all the flows through the ecosystem	[28]
**FD/TST**	Flows to Detritus/TST		Flows to detritus over the sum of all the flows through the ecosystem	[28]
**Q/TST**	Total consumption/TST		Total consumption over the sum of all the flows through the ecosystem	[28]
**R/TST**	Total respiration/TST		Total respiration over the sum of all the flows through the ecosystem	[28]
**Ex/TST**	Total exports/TST		Total exports of the system over the sum of all the flows through the ecosystem	[28]
**PP/P**	PP/Total Production		Primary production over total production	[28]
**MeanPz (MaxPz)**	Mean (Max) proportion of total mortality due to predation		The mean (or Maximum) proportion of each group's total mortality that was accounted for by each predator	[103]
**meanEE**	Mean Ecotrophic Efficiency	%	Ecotrophic efficiency of a group is that proportion of the production that is utilized in the system.	[28]
**TBco**	Total Biomass (excluding first trophic level)	t·km^−2^	Total biomass of the community excluding detritus and primary producers	[28]
**mTLco**	Mean Trophic Level of the Community		Weighted average trophic level for functional groups with a TL>2	[28]
**TEm**	Mean Transfer Efficiency	%	Geometric mean of transfer efficiencies for trophic level II to IV	[33]
**A/C**	Ascendency/Capacity	%	Relative Ascendency, dimensionless index of ascendency - index of organisation of the food web	[45]
**O/C**	Overhead/Capacity	%	Relative overhead, dimensionless index of the ecosystem's strength in reserve	[45]
**IFO**	Internal Flow Overhead or redundancy	%	Indicator of the change in degrees of freedom of the system, or an indicator of the distribution of energy flow pathways in the system	[45], [47], [48]
**FCI**	Finn's Cycling Index	%	Quantifies the relative amount of recycling and is an indication of stress and structural differences either among models [44]	[44]
**SOI**	System Omnivory Index		Variance of trophic levels in the diet	[24], [49]
**KS**	Keystoneness		Index of the ability of a trophic group with low biomass to influence others	[52]
**KD**	Dominance		Index of both high biomass and high influence	[52]
**Fishing indicators**				
**TC**	Total Catches	t·km^−2^·y^−1^	Total landings and discards exported from the system	[28]
**TLc**	Mean Trophic Level of the Catch**		Average trophic level of all caught species using weighted by yield	[50]
**L_index_**	Loss in production Index		Loss in secondary production due to fishing	[28]
**P_sust_**	Probability of being sustainable fished	%	Probability of the system to be sustainably fished adopting [55] ecosystem overfishing definition and criteria	[51], [53], [54]

*Excluding the cases where the indicator was 0 due to no fishing.

This study is among the first to analyse a large variety of EwE models from different systems in an organised and systematic way. It presents the largest meta-analysis of the structural and functional indicators of marine food webs to date and adds to the general theory of marine food web dynamics and its use for ecosystem conservation and management. It also includes statistical analyses to address co-variance of models and issues of structural uncertainty in these models. The statistical analysis makes this work unique, and the study also includes new and advanced analyses on the key species concept.

## Materials and Methods

### a) Food web models

A description of the theory and algorithms behind Ecopath with Ecosim (EwE) is given in File S1. The locations of the ecosystems represented by the 105 published EwE models of marine ecosystems from around the world used in this study are shown in Fig. 2. The meta-data (i.e., ecological, network, and synthetic indicators) used to describe the ecosystems are available in Table S1. The models ranged from very simple, with 6 component nodes or functional groups in the Canary Island [Canary Islands 37] to more complex models consisting of 68 groups in the North Sea [North Sea 38], with an average number of 26 groups. In terms of depth, the systems represented ranged from less than a meter deep in Venice Lagoon [Venice Lagoon 39], to the deep sea off the Cape Verde Islands [40], and the West Coast of Scotland [41]. The timeline of the models spanned from 1880 (North Sea) to various models that include recent data from the 2000s. The models represent ecosystems from all continents (except Antarctica), with 17 African systems, 14 Australasian systems, 35 European systems, 31 North American, and 8 South American systems. Mexico (16) has the most models, followed by Italy (8), Canada (8), and the USA (7). The latitude and location of the model areas and references to the papers describing these models are given in Table S1.

Many of the models were previously developed to achieve a first description of the ecosystems using the mass balance approach (42 models), while some had estimates of the ecological network analysis (ENA) parameters (36 models) or were used to compare the Ecopath approach to inverse methods (5 models) (Table S1). In addition, several models had been developed for theoretical dynamic analysis, spatial analysis, or policy analysis (12 models) while 17 models were fitted to time series to hindcast model dynamics, and 6 were used for policy and fisheries impacts. All models used in this analysis were previously peer-reviewed and published in the primary scientific literature (Table S1).

### b) Ecological food web- and fishing impact indicators

The food web models representing different marine systems (Table S1) were used to analyse ecosystem structure and function patterns by trait. We calculated several ecological indicators from ecological network analysis (ENA) and performed a meta-analysis across ecosystems applying different statistical multivariate approaches. The indicators were chosen because they had previously been widely applied to highlight ecosystem structure and functioning, were thought to be robust to differences in model construction (such as the number of functional groups) and were independent from currency used for biomass and flows (Table 1) [42], [43]. In addition, the robustness of these indicators to model construction was further checked by Factorial Analysis (see section below).

The network analysis indicators are mainly related to the total systems throughput (TST), which is the sum of all flows in the model and considered an overall measure of the “ecological size” of the system [44]:
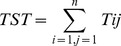
(1)where *T_ij_* is the flow between any two compartments and includes respiration and export flows.

The more descriptive indicators include the primary production/total systems throughput (PP/TST), flow to detritus/TST (FD/TST), total consumption/TST (Q/TST), total respiration/TST (R/TST), total exports/TST (Ex/TST), total biomass excluding detritus (TBco), and the ratio between primary production and total production (PP/P).

The development capacity (C) of the system is the thermodynamic limit of growth in the system and is calculated as: 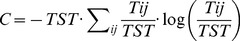
(2)


It scales the TST to a measure of the information carried by flows. Capacity is divided between ascendency (A) and the overhead (O). Ascendency is an index of the organisation of a food web [45] and is defined in terms of flow:
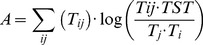
(3)and overhead (O, an indicator of the ecosystem's strength in reserve [45]) is calculated as:



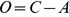
(4)Overhead and Ascendency are divided into export, dissipation and internal flows [46], and the overhead on internal flows (IFO) has been used as an index of ecosystem redundancy [47]. The redundancy is an indicator of the change in degrees of freedom of the system, or the distribution of energy flow among the pathways in the ecosystem [48], and is calculated as:
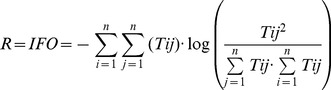
(5)


The Finn Cycling Index (FCI) quantifies the amount of recycling relative to TST and is an indication of stress and structural differences [44], and is calculated as:
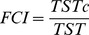
(6)where TST_c_ is the total flow that is recycled.

Other ecological indicators are related to the trophic level (TL) concept, which is the average number of steps for energy to move from primary producers to higher-level consumers and provides an indication of the trophic position of an organism. Thus for a given predator *j* the TL is calculated as:
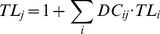
(7)where *TL_i_* is the trophic level of prey *i* and *DC_ij_* is the proportion of prey *i* in the diet of predator *j*. The mean trophic level of the community (mTLco) is calculated as the weighted average TL for functional groups with TL>2, as:
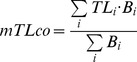
(8)where the weighting factor is the biomass of each functional group (Bi). TL>2 is used to reduce the variability in terms of biomass and production that comes with using low trophic levels.

The system's omnivory index (SOI) is defined on the basis of the omnivory index (OI) of each food web component. It indicates the variance of trophic levels in the diet, and is:
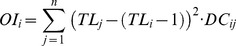
(9)


So from the OI of each functional group, the SOI for the food web is calculated as:
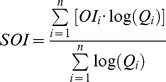
(10)where Q_i_ is the food intake of each consumer [24], [49].

The mean transfer efficiency (TEm) for the food web was calculated as the geometric mean of transfer efficiencies for each of the integer trophic levels II to IV. The transfer efficiency of a trophic level is calculated as the sum of the flow transferred from any given level to the next higher level, plus exports (e.g., catches) from the given level relative to the input (or throughput) of the given trophic level.

We also included indicators measuring fishing intensity and impacts in the ecosystems: the mean trophic level of the catch (TLc) [TLc, 50], the primary production required to sustain the catches [PPRc, 28], the loss in production index (L_index_) [L index, 51] and the probability of an ecosystem being sustainably fished P_sust_ [Psust, 52].

Similar to the mTLco, the mean trophic level of the catches (TLc) is calculated as the weighted average of TL of caught species using catches (*Y_i_*) as the weighting factors:
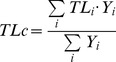
(11)


The proportion of primary production required for the exploited fishery's catch (PPRc) is defined as:
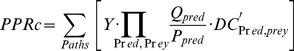
(12)where *P* is production, *Q* consumption, and *DC′* is the diet composition for each predator/prey interaction in each path from primary production or detritus through the food web to the catch, with cycles removed from the diet compositions [28]. PPRc can be expressed in percentage terms when it is normalized with the primary production (PPRc/PP  =  PPRc%).

The Loss in Production Index (L_index_) and the probability for the ecosystem to be sustainably fished (p_sust_) are both used to evaluate the ecosystem effects of fishing [51], [53], [54]. The probability of being sustainably fished was defined by adopting Murawski's [55] ecosystem overfishing definition and criteria, and it includes structural and functional degradation associated with stock collapses and overexploitation of marine resources. L_index_ is defined as: 
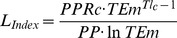
(13)where the loss in production is estimated on the basis of TLc, TEm and PPRc, that is compared to the primary production at the base of the food web (PP). The probability that such energy loss is sustainable was calculated by comparing L_index_ for a set of overexploited and sustainably exploited ecosystems as reported in Libralato et al. [51]. This allows the definition of a non-linear empirical relationship between the L_index_ and P_sust_ to be used for calculating sustainability of fisheries.

### c) Factorial analysis

To analyse marine food webs by the ecological and fishing indicators described above, 7 traits (factors) were chosen: ecosystem type, latitude, ocean basin, depth, size, time period, and exploitation of the ecosystem represented by the model.

Size of ecosystem included six classes (1–10 km^2^, 11–100 km^2^, 101–1,000 km^2^, 1,001–10,000 km^2^, 10,001–100,000 km^2^, >100,000 km^2^), depth included seven classes (<5 m, <10 m, <20 m, <50 m, <100 m, <200 m, >200 m), and ecosystem type was divided into six classes (lagoon, estuary, bay, coastal, reef, continental shelf, and upper slope). The classes were developed following from previous analyses [56], [57] and were included where there were enough models per class (n>5). The modelled food webs were divided into four latitude classes from the equator to the poles (15°S–15°N and 15–30°, 30–60°, 60–90°, north and south combined). The food webs were also divided from west to east into seven ocean basin classes following a longitudinal gradient (West Atlantic, East Atlantic, North and Baltic Sea, Mediterranean, Indian, West Pacific, East Pacific). The time periods represented by the models were divided into four classes: before 1970, 1970–1980, 1980–1990 and after 1990. With regards to exploitation, the ecosystems were split into three classes: high exploitation, low exploitation (including food webs with recreational fishing only) and no exploitation.

Significant differences between ecosystems were assessed first by comparing all ecological indicators and then comparing individual indicators with each trait and using the non-parametric multivariate permutational analysis of variance (PERMANOVA, in PRIMER with PERMANOVA+ v. 6, PRIMER-E Ltd., Plymouth, UK) on the Euclidean distance matrix. PERMANOVA calculates a pseudo-F statistic that is analogous to the construction of the traditional F-statistic for multifactorial univariate ANOVA models, but uses permutation procedures (here 9999 permutations) to obtain p-values for each term in the ANOVA model [58]. Due to the limited number of observations, and an unbalanced design among traits, we performed a 1-way analysis by trait using first all the indicators together and then each indicator separately. Even if we used indicators less affected by model construction [42], [43], the number of functional groups and aggregation used to represent food webs can still influence model outputs and analyses. Therefore we assessed the robustness of indicators to model configuration by including 3 factors as covariates in the PERMANOVA analysis: (i) the number of functional groups or nodes of each food web model; (ii) the number of living groups; and (iii) the total number of trophic links. We used a Type I (or sequential) partition of the sum of squares (SS) since individual SS terms were not independent when including covariates.

When significant, the variation of the different ecological food web indicators and fishing indicators by trait (ecosystem type, latitude, ocean basin, depth, size, time period, and exploitation was plotted using boxplots.

### d) Key functional groups

Key functional groups are those with important roles in the food web, and include keystone and structuring functional groups [59]. Keystone groups have relatively low biomass but disproportionately large effects on the food web [52], [59], while structuring groups have large effects due to their relatively high biomass [60].

The marine food web models were used to calculate the absolute overall effect of a species on the food web as the sum of all the direct and indirect effects, quantified through the mixed trophic impact analysis (MTI). The MTI analysis quantifies the direct and indirect impacts that each (impacting) group has on any (impacted) group of the food web [61]. The absolute overall effect was then compared with the biomass proportion of each group to identify key species: either keystone (low biomass proportion and high overall effect) or key structuring groups (high biomass proportion and high overall effect [59]). The role of functional groups was assessed through the key role (keystone or structuring functional groups), trophic level (TL) and proportion of top down effects (td; where bottom-up effect is calculated as bu  = 100 – td).

On the basis of the work done by Libralato et al. [52], the overall effect of each functional group *i* on the ecosystem was estimated through:
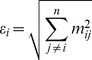
(14)in which the effect of the change in biomass on the group itself (i.e., *m_ii_*) is not included. Moreover, accounting for the fraction of positive and negative contributions to the overall effect allows evidence for contribution of bottom-up (positive *m_ij_*) and top-down (negative *m_ij_*) effects [52]. This was synthesized here by reporting the fraction of top-down contributions (td, as percentage) to the total effect.

The overall effect of each functional group on the ecosystem combined with information on the group's density is useful to identify its role in the ecosystem and to identify key functional groups [59], [60]. In particular, groups with a high impact might be identified as keystone or dominant groups if they have a low or high biomass in the ecosystem, respectively [59]. The biomass was found by calculating the contribution of each functional group to the total biomass of the food web, as:
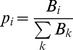
(15)where *p_i_* is the biomass proportion for group *i*; see Libralato et al. [52].

Using the definition given by Libralato et al. [52], the index of keystoneness was calculated as follows:
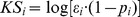
(16)that has the property of attributing high values of keystoneness to functional groups that have both low biomass proportion and high overall effect (as defined above).

The absolute overall effect and the biomass proportion of the functional group were used to identify key functional groups (i.e. species or groups of species with particular roles in the food web), complementing the keystone indicator as proposed by Libralato et al. [52].

The analysis of biomass proportion and overall effect helped to distinguish those key groups that might be difficult to disentangle in terms of keystoneness index only. Therefore, similarly to the keystoneness, an index of dominance of species was calculated from: 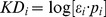
(17)that assumes high values for functional groups that have both high biomass proportion and high overall trophic effect (as defined above): these groups are considered the dominant functional groups, also known as structural groups. The overall impact ‘keystoneness’ and ‘dominance’ proposed here were estimated for each living functional group (thus excluding detritus groups) in the suite of food web models.

For each group, the method provided values for KS and KD: generally groups ranking high in KS were ranking low in KD and *vice versa*. In order to simplify the evaluation, analyses were performed on the most influential functional groups identified as the top 5% ranking groups over all the models. Thus, high ranking keystone functional groups were defined with KS ≥0 and dominant species those with KD ≥−0.7.

Significant differences in the proportion of key functional groups (keystone and structural species) in relation to exploitation level and ecosystem traits (ecosystem type, latitude, ocean basin, depth, size, period represented and exploitation) were evaluated using Correspondence analysis (CA) and performed using Statistica version 6.1 (Statsoft; www.statsoft.com) [62]. CA evaluates deviation from independence between key groups' frequency and traits on the basis of Chi-squared statistic, and decomposes the overall Chi-square in contributions from each combination of trait/key groups, see for example [see for example 63,64]. Therefore, on the basis of the main contributors to overall Chi-square, CA identifies the combination of traits that deviates more from the expected values and the complete independency of factors [complete independency of factors 62].

## Results

### a) Ecological food web indicators of marine ecosystems

We calculated several ecological indicators from flows and biomasses of food webs (Table 1).

There were significant differences in the food web properties of marine ecosystems by ecosystem type, ocean basin, depth, size, and whether the ecosystem was fished or not (Table 2, values in bold). These results were robust considering differences in model construction since they were corrected for covariance, or the way food webs were described. In contrast, ecological indicators were maintained in ecosystems over time (with no significant difference in food web properties by year, suggesting that the main past and present configurations of marine food webs prevail) and latitudinal gradient (Table 2).

**Table 2:** Significance between ecosystems indicators when grouping data by ecosystem traits.

	TRAITS						
Indicators	Ecosystem type	Latitude	Ocean	Depth	Size	Year	Fishing
**Ecological indicators**	0.01*	0.16 (Fg, Lg, Tl)	0.00* (Fg, Lg, Tl)	0.02* (Fg, Lg, Tl)	0.00* (Fg, Lg, Tl)	0.60 (Fg, Lg, Tl)	0.02* (Fg, Lg, Tl)
**Fishing indicators** ¥	0.41	0.12	0.17	0.00* (Lg)	0.02* (Lg)	0.33	¥

Analyses were performed with PERMANOVA. *: significant difference of the indicator between levels of the factor. Cells with (Fg, Lg, TI) indicate that the covariate of number of functional groups (Fg), number of living groups (Lg) and number of trophic links (Tl) had a significant effect on the indicator, while cells with (Lg) indicate significant covariance by number of living groups only.

Covariate functional groups (Fg), Covariate number of living groups (Lg), Covariate trophic links (Tl) are reported.

¥ We excluded the impact of fishing on fishing indicators because for some models there was no fishing, thus fishing indicators could not be calculated.

**Figure 3:**
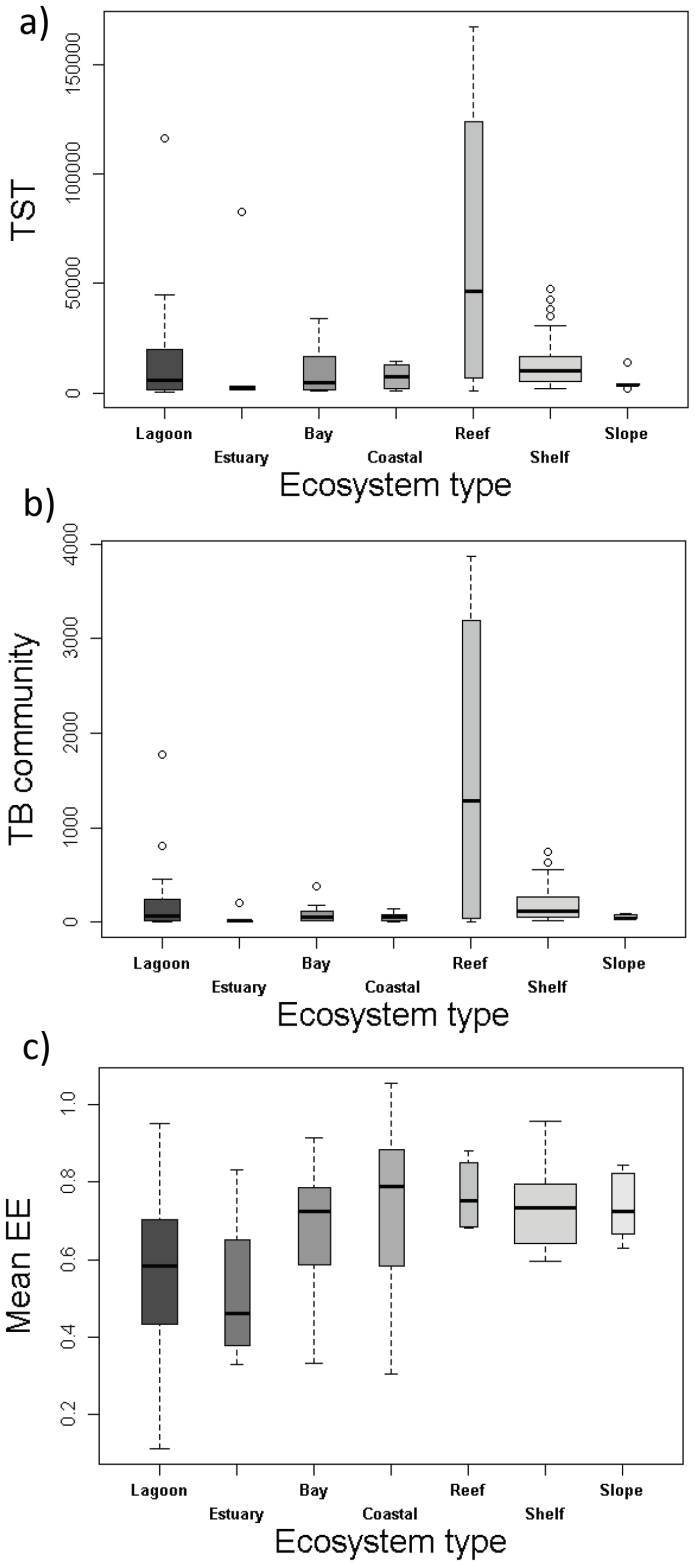
Boxplot of significant differences of food web ecological indicators by ecosystem type. The smallest observation (minimum), lower quartile, median, upper quartile, largest observation (maximum) and outliers are indicated. The boxes are drawn with widths proportional to the square-roots of the number of observations in each class. TST  =  total systems throughput (t.km^−2^.year^−1^), TB community  =  total biomass of the community (t.km^−2^), and mean EE  =  mean ecotrophic efficiency (proportion).

When examining specific ecological indicators by ecosystem type (Table 3), there were significant differences in Total System Throughput (TST, the measure of total trophic flows within an ecosystem) and Total Biomass of the community (TBco), with higher values in reefs, lagoons and shelves (Fig. 3a & 3b). The mean Ecotrophic Efficiency (meanEE, the proportion of overall production used within the system) increased from estuaries to shelves and slopes (Fig. 3c). These results were significant after accounting for covariance.

**Table 3:** Significance of individual indicator differences between levels of ecosystems traits.

	TRAITS																				
Indicators	Ecosystem type			Latitude			Ocean			Depth			Size			Year			Fishing		
	Fg	Lg	Tl	Fg	Lg	Tl	Fg	Lg	Tl	Fg	Lg	Tl	Fg	Lg	Tl	Fg	Lg	Tl	Fg	Lg	Tl
**A/C**	0.68	0.68	0.68	0.13	0.13	0.13	0.01*	0.01*	0.01*	0.49	0.49	0.49	0.35	0.35	0.35	0.63	0.63	0.63	0.47	0.47	0.47
**O/C**	0.60	0.60	0.60	0.11	0.11	0.11	0.00*	0.00*	0.00*	0.53	0.53	0.53	0.22	0.22	0.22	0.75	0.75	0.75	0.57	0.57	0.57
**IFO**	0.42	0.42	0.42	0.09	0.09	0.09	0.00*	0.00*	0.00*	0.81	0.81	0.81	0.16	0.16	0.16	0.73	0.73	0.73	0.70	0.70	0.70
**FCI**	0.14	(0.14)	0.14	0.09	0.09	0.09	0.30	(0.30)	0.30	0.67	0.67	0.67	0.01*	(0.01*)	0.01*	1.00	1.00	1.00	0.14	(0.14)	0.14
**PP/TST**	0.29	0.29	0.29	0.69	0.69	0.69	0.03*	0.03*	0.03*	0.14	0.14	0.14	0.07	0.07	0.07	0.30	0.30	0.30	0.16	0.16	0.16
**FD/TST**	(0.12)	(0.12)	0.12	(0.73)	(0.73)	0.73	(0.00*)	(0.00*)	0.00*	(0.01*)	(0.01*)	0.01*	(0.90)	(0.90)	0.90	(0.85)	(0.85)	0.85	(0.54)	(0.54)	0.54
**SOI**	(0.24)	0.24	0.24	(0.43)	0.43	0.43	(0.01*)	0.01*	0.01*	(0.80)	0.80	0.80	(0.07)	0.07	0.07	(0.42)	0.42	0.42	(0.44)	0.44	0.44
**TB/TST**	(0.52)	0.52	0.52	(0.62)	0.62	0.62	(0.75)	0.75	0.75	(0.39)	0.39	0.39	(0.04*)	0.04*	0.04*	0.50	0.50	0.50	(0.12)	0.12	0.12
**TST**	0.01*	0.01*	0.01*	0.22	0.22	0.22	0.45	0.45	0.45	0.17	0.17	0.17	0.21	0.21	0.21	0.59	0.59	0.59	0.06	0.06	0.06
**Q/TST**	0.43	0.43	0.43	0.29	0.29	0.29	0.01*	0.01*	0.01*	0.13	0.13	0.13	0.45	0.45	0.45	0.60	0.60	0.60	0.35	0.35	0.35
**R/TST**	0.37	0.37	0.37	(0.28)	0.28	0.28	(0.00*)	0.00*	0.00*	(0.01*)	0.01*	0.01*	(0.16)	0.16	0.16	(0.52)	0.52	0.52	(0.48)	0.48	0.48
**Ex/TST**	0.48	0.48	0.48	0.09	0.09	0.09	0.01*	0.01*	0.01*	0.14	0.14	0.14	0.02*	0.02*	0.02*	0.41	0.41	0.41	0.12	0.12	0.12
**PP/P**	(0.19)	(0.19)	0.19	0.24	(0.24)	0.24	(0.29)	(0.29)	0.29	0.86	(0.86)	0.86	(0.21)	(0.21)	0.21	0.21	(0.21)	0.21	(0.05*)	(0.05*)	0.05*
**Mean Pz**	0.88	(0.88)	(0.88)	0.81	(0.81)	(0.81)	0.39	(0.39)	0.39	0.78	(0.78)	(0.78)	0.63	(0.63)	0.63	0.59	(0.59)	(0.59)	0.27	(0.27)	(0.27)
**Max Pz**	(0.92)	(0.92)	(0.92)	(0.47)	(0.47)	(0.47)	(0.02*)	(0.02*)	0.02*	(0.96)	(0.96)	(0.96)	(0.31)	(0.31)	0.31	(0.77)	(0.77)	(0.77)	(0.17)	(0.17)	(0.17)
**Mean EE**	(0.01*)	(0.01*)	0.01*	0.04*	(0.04*)	0.04*	0.23	(0.23)	0.23	0.00*	(0.00*)	0.00*	0.00*	(0.00*)	0.00*	0.71	(0.71)	0.71	0.01*	(0.01*)	0.01*
**Tbco**	0.00*	0.00*	0.00*	0.15	0.15	0.15	0.44	0.44	0.44	0.08	0.08	0.08	0.02*	0.02*	0.02*	0.67	0.67	0.67	0.01*	0.01*	0.01*
**mTLco**	(0.68)	(0.68)	0.68	(0.72)	(0.72)	0.72	(0.05*)	(0.05*)	0.05*	(0.06)	(0.06)	0.06	(0.07)	(0.07)	0.07	(0.02*)	(0.02*)	0.02*	(0.50)	(0.50)	0.50
**TEm**	(0.08)	0.08	0.08	(0.30)	0.30	0.30	(0.03*)	0.03*	0.03*	(0.01*)	(0.01*)	0.01*	(0.17)	0.17	0.17	(0.38)	0.38	0.38	(0.91)	0.91	0.91
**TLc** ¥	0.07	(0.07)	0.07	0.02*	(0.02*)	0.02*	0.01*	(0.01*)	0.01*	0.00*	(0.00*)	0.00*	0.06	(0.06)	0.06	0.77	(0.77)	0.77	¥	(¥)	¥
**TC** ¥	0.22	0.22	0.22	0.61	0.61	0.61	0.28	0.28	0.28	0.00*	0.00*	0.00*	0.02*	0.02*	0.02*	0.92	0.92	0.92	¥	¥	¥
**PPR%** ¥	0.97	0.97	0.97	0.09	0.09	0.09	0.35	0.35	0.35	0.11	0.11	0.11	0.10	0.10	0.10	0.40	0.40	0.40	¥	¥	¥
**L_index_** ¥	0.60	0.60	0.60	0.31	0.31	0.31	0.65	0.65	0.65	(0.03*)	0.03*	0.03*	0.03*	0.03*	0.03*	0.48	0.48	0.48	¥	¥	¥
**P_sust_** ¥	0.83	0.83	0.83	0.49	0.49	0.49	0.45	0.45	0.45	0.07	0.07	0.07	0.45	0.45	0.45	0.02*	0.02*	0.02*	¥	¥	¥

Analysis was perform with PERMANOVA. Vaues with a * indicate a significant difference of the indicator between levels of the factor. Values in () indicate that the covariate had a signficiant effect on the indicator.

Covariate functional groups (Fg), Covariate number of living groups (Lg), Covariate trophic links (Tl).

¥ We excluded the impact of fishing on fishing indicators because for some models there was no fishing, thus fishing indicators could not be calculated in those cases, which means we were not comparing like with like.

**Figure 4:**
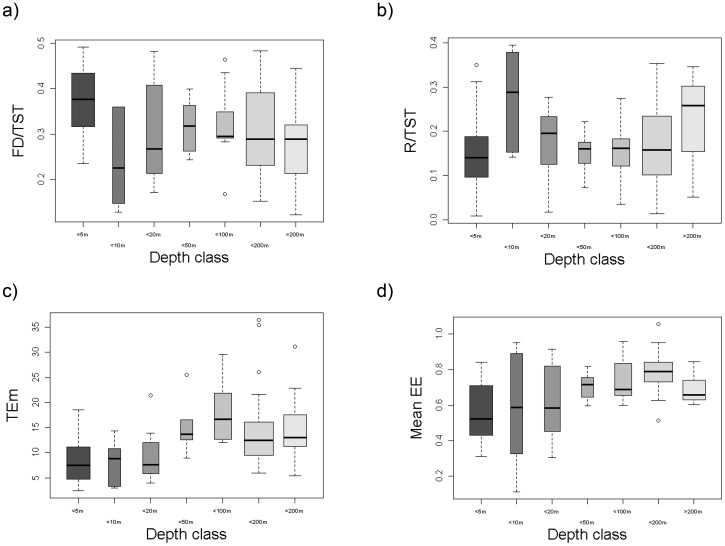
Boxplot of significant differences of food web ecological indicators by depth class. The smallest observation (sample minimum), lower quartile, median, upper quartile, largest observation (sample maximum) and outliers are indicated. The boxes are drawn with widths proportional to the square-roots of the number of observations in each class. FD/TST  =  flow to detritus/total systems throughput (proportion), R/TST  =  respiration/total systems throughput (proportion), TEm  =  mean transfer efficiency (%) and mean EE  =  mean ecotrophic efficiency (proportion).

Some ecological indicators also differed significantly with depth (Table 3), with the Flow to Detritus (FD/TST, the non-living particulate organic matter that returns to the trophic flow as a ratio of the total flow), total Respiration (per unit of trophic flow, R/TST), mean Ecotrophic Efficiency (meanEE) and mean Transfer Efficiency (TEm, or the conversion of production from lower to higher trophic levels) all showing significant differences (Fig. 4). The flow to detritus (FD/TST, Fig. 4a) was higher in shallower systems, with the trend being reversed in respiration (R/TST, Fig. 4b) and mean transfer efficiency (Tem, Fig. 4c). The trend in mean Ecotrophic Efficiency (meanEE, Fig. 4d) also showed correlation with ecosystem type, where shallower systems (such as lagoons, estuaries and bays) had lower mean Ecotrophic Efficiencies than deeper systems (such as shelves and slopes).

**Figure 5:**
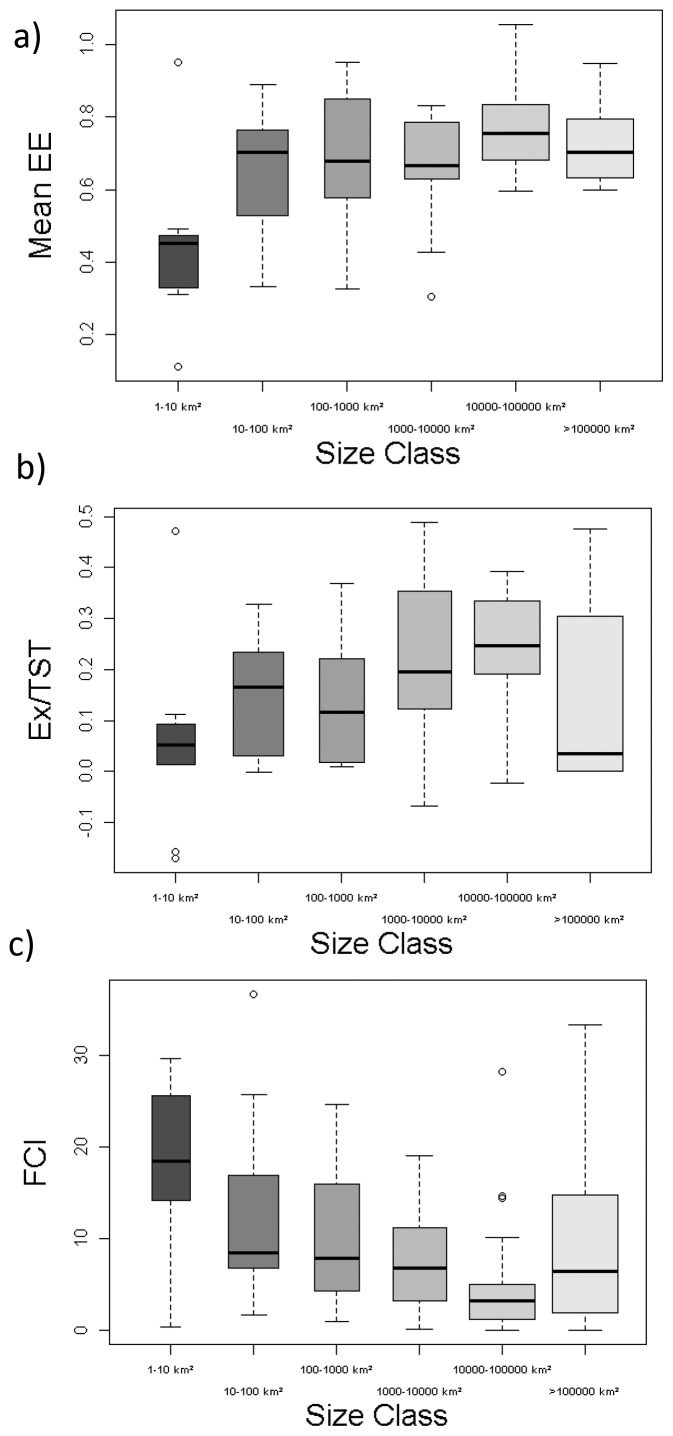
Boxplot of significant differences of food web ecological by size class. The smallest observation (sample minimum), lower quartile, median, upper quartile, largest observation (sample maximum) and outliers are indicated. The boxes are drawn with widths proportional to the square-roots of the number of observations in each class. Mean EE  =  mean ecotrophic efficiency (proportion), Ex/TST  =  export/total systems throughput (proportion) and FCI  =  Finn cycling index.

Similarly, ecological indicators were also significantly different by ecosystem size (Table 3). Again, the mean Ecotrophic Efficiency was lower in smaller systems (meanEE, Fig. 5a), and increased with size, while the export from the system (export of matter per unit of flow, Ex/TST) was also lowest in small systems and increased in larger systems (Fig. 5b). This was converse to the trend in the Finn Cycling index (FCI, an index of the relative amount of recycling in the ecosystem), which was highest in smaller systems (Fig. 5c) and declined as size increased.

**Figure 6:**
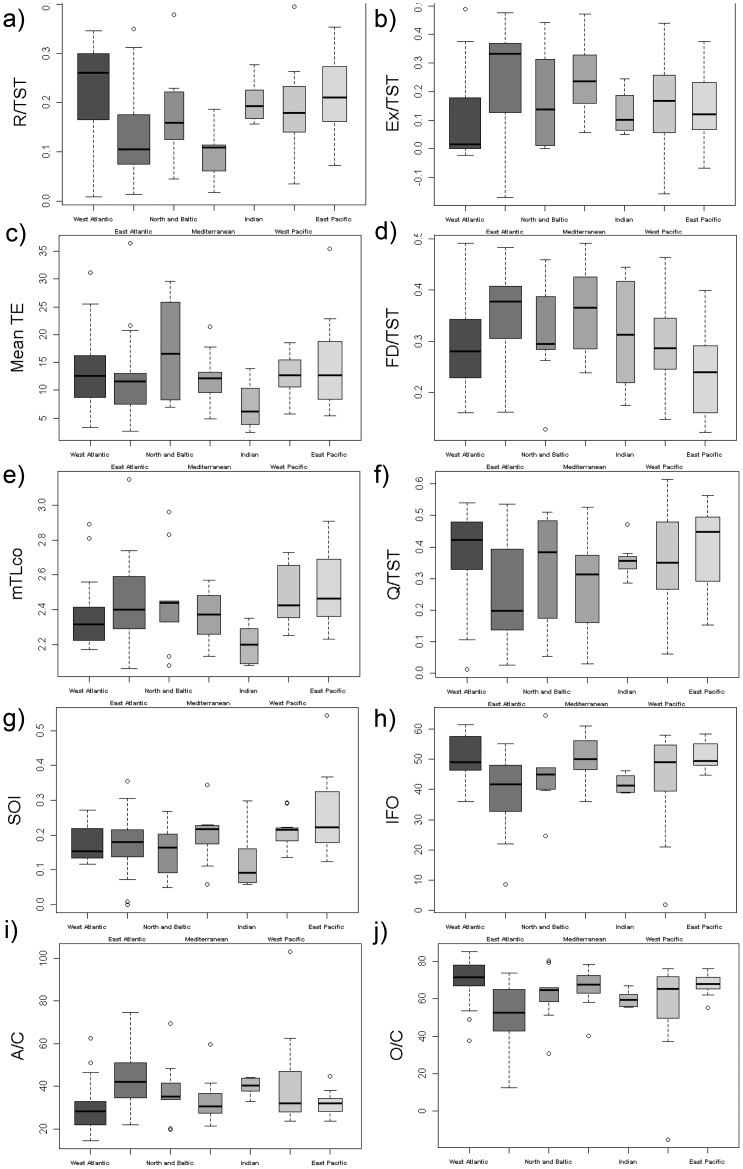
Boxplot of significant differences of food web ecological by ocean basin. The smallest observation (sample minimum), lower quartile, median, upper quartile, largest observation (sample maximum) and outliers are indicated. The boxes are drawn with widths proportional to the square-roots of the number of observations in each class. R/TST  =  respiration/total systems throughput, Ex/TST  =  export/total systems throughput (proportion), mean TE  =  mean transfer efficiency, FD/TST  =  flow to detritus/total systems throughput (proportion), MTLco  =  mean trophic level of the community, Q/TST  =  consumption/total systems throughput (proportion), SOI  =  systems omnivory index, IFO  =  Internal flow overhead (%), A/C  =  relative ascendency (%), O/C  =  relative overhead (%).

The ecosystem trait that showed significant differences for most ecological indicators was ocean basin (Ocean, Table 3). Respiration (R/TST, Fig. 6a), export of matter per unit of flow (Ex/TST, Fig. 6b), mean transfer efficiency (TEm, Fig. 6c), flow to detritus (FD/TST, Fig. 6d), the mean trophic level of the community (mTLco, the average trophic level for functional groups with a TL>2 that represents a mean trophic position of organisms in the community, Fig. 6e), total consumption (per unit of flow, Q/TST, Fig. 6f), system omnivory index (SOI, the variance of trophic levels in the diet, Fig. 6g), redundancy or internal flow overhead (IFO, the distribution of energy flow pathways in the system, Fig. 6h), relative ascendency (A/C, an index of organisation of the food web, C being the Development Capacity of the system, Fig. 6i) and relative overhead (O/C, the index of the ecosystem's strength in reserve, Fig. 6j) all showed significant changes with ocean basin. A general decreasing trend between the West and East Atlantic (and the reverse in the West and East Pacific) was observed in respiration (R/TST), consumption (Q/TST) and the redundancy (IFO) in these systems, while an increasing trend was shown in the flow to detritus (FD/TST) and the relative ascendency (A/C). It was also clear that some differences between Ocean basins were reproduced in ecosystem indicators such as the mean transfer efficiency (TEm), mean trophic level of the community (mTLco), and the system omnivory index (SOI): the Pacific Ocean had higher values than the Atlantic and Indian Oceans, and the Indian Ocean generally had the lowest value for all those indicators.

Overall, the traits that showed significant differences between model indicators were ecosystem type, latitude, ocean basin, size, and year. Those indicators that were least prone to co-vary with factors (i.e. functional groups, number of living groups, and number of trophic links) were total export (Ex/TST) and total biomass of the community (TBco, showing no differences in cell colour between covariance in Table 3). Both were only significantly different in two traits each (Ex/TST  =  ocean basin and size, TBco  =  ecosystem type and size). Of those ecological indicators analysed, eight were robust to covariates, namely relative ascendency (A/C), relative overhead (O/C), redundancy (IFO), primary production (PP/TST), total systems throughput (TST), consumption (Q/TST), export (Ex/TST), and total biomass of the community (TBco, as highlighted in Table 3). These indicators may be of special interest for future food web studies if correction for covariance is not possible.

### b) Key functional groups of food webs

**Figure 7:**
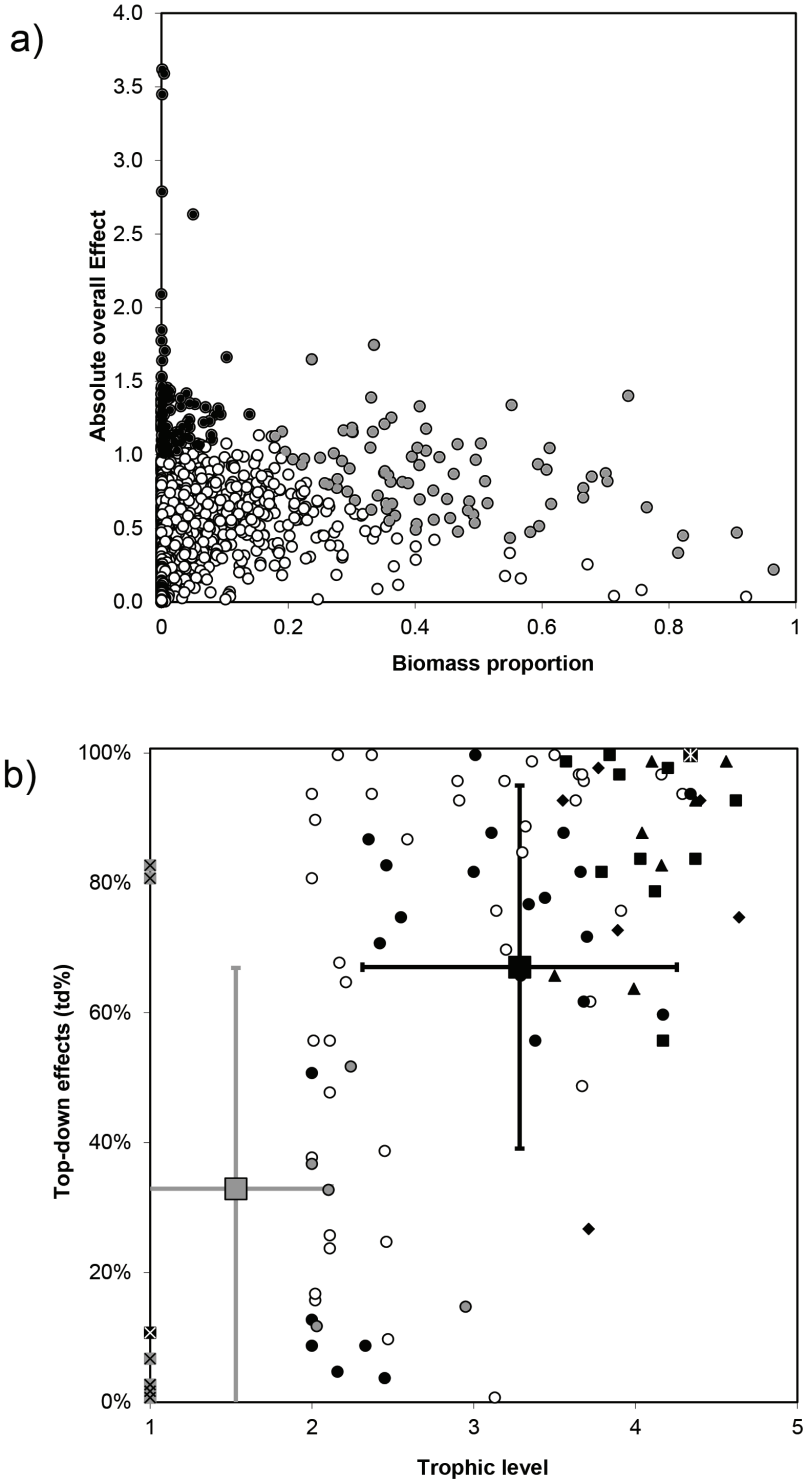
Key ecological roles of functional groups of marine food web models. a) Keystone (KS ≥0; black circles) and dominant groups (KD ≥0.7; grey circles), respectively, in terms of absolute overall effect (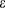) for each food web. Open dots represent non key functional groups. b) The 105 trophic groups franking first in terms of absolute overall effect within each food web model (Fig. 2, Table S1). The figure shows the trophic level (TL) vs. the fraction of top-down effect (td%). Groups identified as keystones are represented in black symbols and dominant groups are reported in grey symbols, respectively, whereas open circles represent non key functional groups. Groups are highlighted for both keystones and dominant: birds (star within square), marine mammals (triangles), sharks and rays (squares), top-predators (romboid), primary producers (crossed squares), other groups (circles). Large squares with error bars identify mean+/−SD for all keystones and dominants identified in the 105 models.

Keystone and key dominant/structuring group indicators have been estimated for all the 2,635 functional groups in the 105 models, and properties of keystone and key dominant/structuring groups were defined on the basis of the top 5% of the ranking groups. The top ranking keystone functional groups had an average TL of 3.28±0.97, an average biomass proportion of 0.022±0.044, and a clear prevalence of top-down effects with an average td effect of 67%±28%. Conversely, top ranking key structuring functional groups had an average TL of 1.53±0.6, an average biomass proportion of 0.43±0.17 and an average td effect of 33%±34% (Fig. 7a & 7b). The last result highlighted a prevalence of bottom-up effects in key structuring groups. However, the large range might be due to predatory groups having high td (i.e. benthic invertebrates), and therefore assumptions about bottom-up effects as a characteristic feature should be made with caution.

The groups' ranking in terms of overall effect from each of the 105 food webs showed that several groups were identified to be keystones (i.e. with low biomass proportion; Fig. 7b, black symbols) and a large proportion of these groups had high trophic levels. Smaller organisms were prevalently key structuring groups (i.e. with high biomass proportion and high impact; grey symbols). The groups that ranked first in terms of overall effect generally had high trophic levels and many of them were larger organisms such as sharks and rays (ten models, n = 10), top predatory fishes (n = 6), marine mammals (n = 7) and seabirds (n = 1). Producers, and especially benthic primary producers such as macroalgae and pleustophytes, were key structuring species (n = 10). Our results highlight groups whose changes in biomass have the largest effect on the food web, and the main distinctive factor between structuring and keystone species is their biomass proportion, although, this is not surprising as biomass proportion is explicit in the definition of key species.

**Figure 8:**
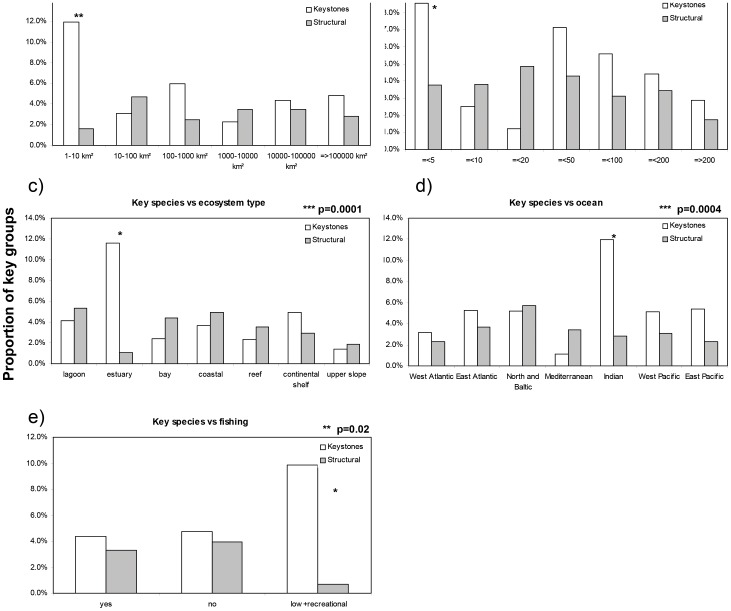
Proportion of key functional groups by food web ecological traits and by exploitation. Graphs report proportion of Keystone (KS ≥0) and dominant groups (KD ≥0.7) by a) ecosystem size, b) depth, c) ecosystem type, d) ocean basin and e) fishing category. Only traits showing significantly different patterns (on the basis of total Chi-squared) are reported (***, ** respectively p<0.01, p<0.05). Main contribution to Chi-squared are highlighted by asterisk (see also Table 4).

Correspondence analysis indicated that the proportion of keystone and structural functional groups showed significant variation with size, depth, and type of ecosystem at p<0.001, as well as significant variability with ocean basin at p<0.001 (Fig. 8 and Table 4). In particular, a significantly higher proportion (with the highest Chi-square values by trait) of keystone groups was found in smaller (size <10 km^2^, Fig. 8a) and shallower (depth ≤5 m, Fig. 8b) ecosystems, in estuaries (Fig. 8c) and in the Indian Ocean models (Fig. 8d). This last result is due to the fact that the Indian Ocean models mostly represent bays and estuaries (Fig. 8 & Table 4). The proportion of structural groups never showed high Chi-squared contributions, thus indicating that dominant species did not distribute differently among traits and have a similar ecological role.

**Table 4:** Chi-squared obtained from the Correspondence Analysis between traits (fishing, size, ecosystem type, latitude, longitude, depth and year) and key functional groups.

Traits	Classes	Key functional group			total Chi-square	p-value	
		Keystones	Structuring	Other			
fishing (df = 4)	no	0.000	0.278	0.010			
	low/recreational	**{8.013}**	2.645	0.116			
	yes	0.484	0.076	0.011	**11.634**	**0.0203**	**
size (df = 10)	1–10 km^2^	**{20.556}**	1.298	0.664			
	10–100 km^2^	1.386	2.003	0.000			
	100–1000 km^2^	0.654	0.263	0.008			
	1000–10000 km^2^	5.163	0.185	0.189			
	10000–100000 km^2^	0.186	0.132	0.001			
	= >100000 km^2^	0.014	0.430	0.009	**33.142**	**0.0003**	***
ecosystem type (df = 12)	lagoon	0.163	4.073	0.078			
	estuary	**{19.073}**	2.589	0.478			
	bay	2.273	1.046	0.023			
	coastal	0.364	1.690	0.011			
	reef	1.000	0.048	0.035			
	continental shelf	0.203	0.549	0.001			
	upper slope	4.964	1.062	0.480	**40.201**	**0.0001**	***
latitude (df = 6)	= <15	0.105	2.491	0.047			
	15–30	2.191	1.221	0.289			
	30–60	0.903	2.399	0.249			
	= >60	0.100	0.299	0.001	10.294	0.113	
Ocean basin (df = 12)	West Atlantic	2.800	0.148	0.201			
	East Atlantic	0.452	0.054	0.038			
	North and Baltic	0.093	0.000	0.005			
	Mediterranean	9.619	0.088	0.418			
	Indian	**{19.528}**	0.042	0.924			
	West Pacific	0.125	0.098	0.019			
	East Pacific	0.373	0.078	0.008	**35.109**	**0.0004**	***
depth (df = 12)	= <5	**{12.451}**	0.552	0.872			
	= <10	0.794	0.119	0.019			
	= <20	4.280	1.599	0.055			
	= <50	1.767	0.620	0.198			
	= <100	0.554	0.000	0.029			
	= <200	0.152	0.027	0.003			
	= >200	4.516	3.735	0.698	**33.038**	**0.0010**	***
year (df = 6)	<1970	0.016	1.287	0.032			
	1970–1980	0.152	1.947	0.119			
	1980–1990	3.219	0.466	0.078			
	>1990	2.775	0.020	0.123	10.235	0.1151	

Between parenthesis the degrees of freedom of Covariance analysis (df). Significant total Chi-squared are bold (*** and ** indicate p<0.01 and p<0.05 respectively). Main contributors to Chi-squared indicated by {}.

### c) The impacts of fishing on marine food web indicators

**Figure 9:**
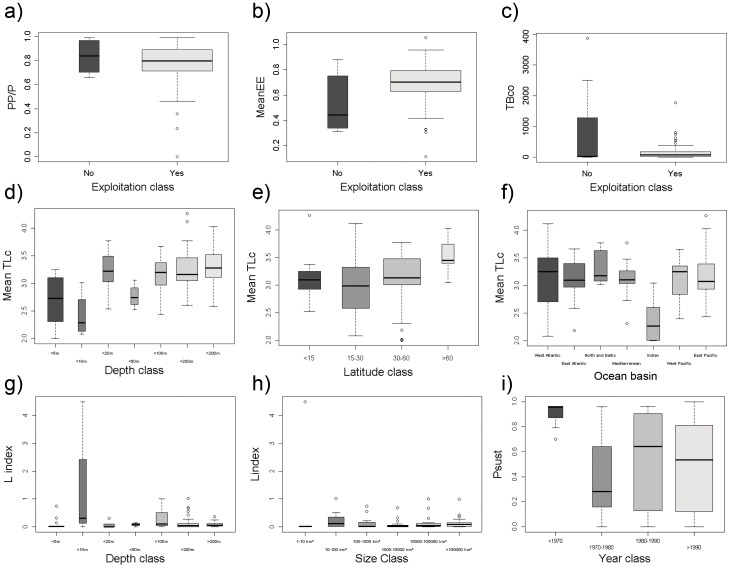
Boxplot of significant differences of food web ecological traits by exploitation and of fishing indicators. The smallest observation (sample minimum), lower quartile, median, upper quartile, largest observation (sample maximum) and outliers are indicated. The boxes are drawn with widths proportional to the square-roots of the number of observations in each class. PP/P  =  primary production/total production (proportion), meanEE  =  mean ecotrophic efficiency (proportion), TBco  =  total biomass of the community (t.km^−2^), Mean TLc  =  mean trophic level of the catch, L_index_  =  loss in production index, and P_sust_  =  probability of being sustainably fished.

Fishing is at present the main human factor that impacts marine food webs [2], [65], [66]. Therefore, this study tested if ecological food web indicators also varied among different exploitation levels in marine ecosystems. Our results showed that primary production (PP/P, the unit of primary production over total ecosystem production), mean Ecotrophic Efficiency (MeanEE) and total community biomass (TBco) showed significant differences between exploited and non-exploited food webs (Table 3; Fig. 9a, 9b & 9c).

Moreover, marine food webs also showed differences in the levels of fishing between systems of different depth and size (Table 2), implying different levels of exploitation strategies and impact from fishing with regard to these ecosystem features. Shallower and smaller ecosystems are closer to the coast, thus in general more exposed to fishing (in some cases for centuries), whereas deep and large ecosystems are less accessible, constituting refuges from fishing. The results show, for instance, the mean trophic level of the catch (TLc, which quantifies the mean trophic position of exploited organisms) increased with depth (Fig. 9d). The trophic level of the catch also increased with latitude (TLc, Fig. 9e) and was lowest in the Indian Ocean and highest in the West Atlantic (Fig. 9f), although the TLc was also high in the North and Baltic Seas. Fishing intensity changed with time and we observed a decrease of the probability of being sustainably fished (P_sust_, Fig. 9i) over time.

Top ranking keystone and structuring species (95^th^ percentile in the KS and KD distribution) were tested for variance according to fishing intensity by analyzing the proportion of these key groups in food webs representing fished, non-fished, or slightly fished (i.e. marine protected areas that included only recreational or artisanal fishing) ecosystems. Of the 2,366 functional groups from the fished ecosystem models, 104 (4.4%) were top ranking keystone groups [52] and 78 (3.3%) were top ranking structuring functional groups. Similar proportions were identified for the non-fished ecosystems: among the 127 functional groups, 6 (4.7%) and 5 (3.9%) groups were identified as top ranking keystone and structural functional groups, respectively. Interestingly, in the 142 functional groups of the lightly exploited food webs, 14 (9.9%) were keystones and only 1 (0.7%) was a structuring functional group. Correspondence analysis between key species and fishing trait was significant at p = 0.02 (Chi-squared  = 11.6; df = 4), with a major contribution from the increase of groups with high keystoneness in lightly exploited food webs (Fig. 8e & Table 4).

## Discussion

Our approach emphasizes the power of using a large database of ecosystem models to quantify food web indicators at the global level. The ability to generalize from different ecosystems constructed using a common EwE approach is one advantage of having comparable models, based on the same framework.

This study provides a comprehensive analysis for how ecosystems function to show how these indicators might be modified in future with additional human impacts such as climate change, eutrophication, acidification, etc. The impact of model structure and its link with model construction are taken into account by using statistical analyses of co-variance, placing this study among the first to analyse a large variety of EwE models from different systems in an organized, systematic and statistical way.

### a) Caveats

This analysis is predicated on a modelling technique that has, like other modelling techniques, its own drawbacks, such as the lack of uncertainty testing in model inputs [67], the difficulty of including non-trophic interactions, and the fact that it does not handle migratory species particularly well [68]. The quality of input data also affects the predictive quality of the outputs. In addition, the suite of models included here range from very early models (created in the early 1990s) with fewer trophic groups, to newer and/or larger models, i.e. models that are more well defined, and therefore have more trophic groups (this is taken into account in our covariance analysis).

At the same time, even models with the same number of trophic groups might have very different topologies; one model with 20 functional groups might have all the herbivorous species combined in one functional group and the top predators defined by species, while another with 20 groups might have one top predator functional group including very different species, and the lower trophic levels defined in more detail. Although this is to some degree encapsulated in the Finn Cycling Index and the Internal Flow Overhead (and this study tested for co-variance in number of trophic links) this still needs to be tested further. The lack of consistency in model construction can be fully addressed only in studies where all models are developed in a comparable and standardized way, as was done in a few other instances [47], [69]. However a large scale study such as ours would be very difficult to achieve using those methods.

In addition, only 8 of the models used here did not include fishing. EwE models and -up effects such as eutrophication and environmental changes are not always represented. These drivers might have been included in the models that were fitted to time series (see Table S1 for details), but was not the main drivers for most of the models used. Thus, top-down and bottom-up drivers are not equally represented in our study, resulting in underrepresentation of the impact of bottom-up drivers on the ecosystem. Specifically, very few models had the microbial loop defined, and the impact of detritus and the detritivorous food chain is underrepresented. Future work should include a study of models where the microbial loop is defined to elucidate the importance of this underrepresented part of the food web.

Furthermore, not all indicators are equally robust. Fulton et al. [70] found that some throughput-based indicators are very useful if one has good diet data and knowledge of the ecosystem structure, and similarly trophic level-based indicators are only effective if one has good diet data. They also suggest that the network indicators such as relative ascendency are dependent on the data and model formulation as well as on reliable knowledge of ecosystem structure, concluding that these indicators would be most useful in well-studied systems. Although this large meta-analysis allowed us to disentangle the effects of each trait and covariates, more data will result in better models, and well-validated models will give more reliable indicators. Our study concurs with that of Fulton et al. [70], by showing that not all indicators can be used without taking account of the number of functional groups, the number of living groups or the number of trophic links (Table 3).

Lassen et al. [71] showed that some indicators obtained from EwE models (biomass of important species, food web productivity, etc.) are useful as indicators of good environmental status for the EU Marine Strategy Framework Directive (MSFD) [72]. In addition to the indicators described by Lassen et al. [71], other ecological indicators used here to describe marine food webs have the potential to be indicators of good environmental status as requested by international law and directives such as the MSFD [72], given their capabilities to describe food web changes and sensitivity to fishing. However, our results highlight that some ecosystem indicators vary by ecosystem trait (depth, type, size, etc.), thus implying that the trait effect needs to be accounted for when setting reference levels and thresholds for conservation and management. If food web indicators vary with ecosystem traits, the process of defining and quantifying reference levels and thresholds [73]–[75] will have to take this into account. Otherwise, management advice could be based on the wrong indicator or on a wrong reference level, or the indicator might be insensitive to the adopted management policies. Our results, therefore, represent a starting point for disentangling the variability of indicators due to ecosystem traits caused by other stressors of interest to managers. For instance, knowing that an indicator is intrinsically lower or higher in certain ecosystem types might help to better understand the locally provided estimates and adapt reference levels and thresholds.

### b) Structure and functioning of marine food webs

Our detailed results showed some interesting general trends. For example, coral reefs seem to have the largest energy flow and the largest total biomass per unit of surface area, and as the included reef systems covered large areas [76]–[79], their mean Ecotrophic Efficiencies were also high. However, the smaller, shallower systems such as lagoons, estuaries, and bays had lower mean Ecotrophic Efficiencies. These smaller systems usually have shorter residence times, with a larger proportion of species that either migrate in and out of the system, and so might not be utilized in the system [80], thus increasing the unexplained mortality in that system, and therefore reducing the mean Ecotrophic Efficiency. The shallower systems also had more flow to detritus, which together with the fact that these systems often included more benthic interactions usually at low efficiency [80], explained the low mean transfer efficiency found for the shallower areas [81].

Systems with high transfer efficiencies often have fewer pathways between trophic levels, while systems such as lagoons, estuaries and bays often have more species at the lower trophic levels – detritivores, suspension feeders, etc., therefore reducing the mean transfer efficiency [82]–[85]. This was also seen in the higher Finn cycling indices in these systems where more energy was recycled [86], while the total export from these systems was significantly reduced. Systems with higher Finn cycling indices often have the ability to recover from perturbations quicker [86], and could therefore be more stable. Conversely, upwelling or pelagic ecosystems, which tend to be deeper, would have higher transfer efficiencies, more export and less recycling [86] and they are often characterised by large fluctuations [83], [84], [87], although this is not specified in our study and should be examined in future.

Interesting results emerged from the comparisons between ocean basins: there were differences between the eastern and western parts of the Atlantic and Pacific Oceans, which were most noticeable in the Atlantic. From the Western to Eastern Atlantic, we found increased flows to detritus, which was reflected by an increase in ascendency and mean trophic level of the community, while the inverse trend was found in respiration, consumption, redundancy, and overhead. These changes were not necessarily due to differences in ecosystem type, depth, or size categories, as these were all similar for the Eastern and Western Atlantic, nor due to differences in the numbers of groups (average number of groups in the Western Atlantic  = 25, Eastern Atlantic  = 29). The difference in overhead and redundancy indicates that the Western Atlantic systems seem to have more “strength in reserve” and that the energy in these systems has more pathways to travel from primary producers to top predators than in the systems of the Eastern Atlantic. The differences in the Atlantic could also be due to differences in the biological carbon pumps in these two systems. Helmke et al. [88] showed that there were higher nutrient inputs in the Western Atlantic and more pulsed production events, which accounted for more carbon being produced and fluxed in the west. This higher production and flux explain the higher consumption, respiration, and overhead in that system compared to the East Atlantic.

The difference between the east and west was reversed to some extent in the Pacific, with a higher flow to detritus and lower respiration and consumption in the west than the east. The Pacific models also have similar numbers of compartments (26 for both Eastern and Western Pacific on average), but the Western Pacific models were mostly large shallow systems including reefs and bays, while in the east, models included more deep continental shelves but also estuaries, bays and lagoons. Thus, these differences in the types of systems, depth, and size (which were more similar in the Atlantic) confounded the interpretation as depth, size, and ecosystem type have an impact on ecosystem indicators. Thus, even though the primary production is higher in all four eastern boundary current systems (California and Humboldt in the Pacific and Canary and Benguela in the Atlantic) [89], the increase it created in the ascendency, flow to detritus and export in the Atlantic was not seen in the Pacific. This confirms the results of Carr and Kearns [89], who found that the increase in primary production did not increase the biomass sustained by the available nutrients as much in the Pacific as it did in the Atlantic. They found that the higher iron content, increased physical retention, and differences in plankton community structure accounted for the higher sustained biomass in the Atlantic eastern boundary current (our Western Atlantic), indicated by a higher redundancy (or internal flow overhead, IOF, Fig. 6h).

The mean trophic level of the community and omnivory showed a general increase from the Atlantic westwards to the Pacific, with only the Indian Ocean having much lower values for these indicators. The Indian Ocean models were mainly bays and estuaries, thus mostly shallow and small, with the lowest mean transfer efficiency and high ascendency. These ecosystems in the Indian Ocean are thus rather inefficient in transferring energy up the food chain, with very low omnivory but high organisation of the food webs. In contrast, the models of the North and Baltic Seas have the highest transfer efficiencies, but also have high ascendency, and are thus most efficient at transferring energy up the trophic chain while also being a well-organised system. The high transfer efficiency in the North and Baltic Seas is probably due to the lower species diversity in the Baltic Sea model areas [90], which has translated into high transfer efficiency between fewer species.

Most ecological food web indicators did not show significant differences over time, thus can be considered invariant properties of ecosystems over the broad time ranges and global scales used in our study. Exceptions were the mean trophic level of the consumer community and the probability of the system to be sustainably fished, which both decreased with time. The probability of being sustainably fished was defined by adopting Murawski's [55] ecosystem overfishing definition and criteria, which includes structural and functional degradation associated with stock collapses and overexploitation of marine resources, whereas in sustainably fished ecosystems the main structure and function are preserved. Thus, from our analyses the most relevant changes on these systems through time were due to fishing, which gives such a strong, global signal as to be detectable using these broad time ranges and global scales, supporting claims that fishing is currently the main impacting human factor on marine food webs [2], [65], [66], [91].

This shows that while ecosystems have stable intrinsic properties, human impacts could still be important. However, we did not test in detail for bottom-up processes such as eutrophication and other environmental drivers; therefore these cannot be excluded as important anthropogenic impacts on marine ecosystems. In addition, our indicator of time was relatively crude, by grouping the models in three periods – based on the year(s) that the models represented. To fully take account of changes, a dynamic analysis of the indicators over time should be performed for each calibrated model. Such evaluations have been done for some indicators, e.g. by Heymans et al. [47] and Tomczak et al. [92], but could not be implemented here for all models as it needs models validated with time series analyses. Nevertheless, the analysis conducted by broad time periods at the global scale has the advantage that it identifies important trends at a global level, disregarding local peculiarities that can be studied only when calibrated dynamic simulations are available.

Testing for changes in ecological food web indicators between exploited and non-exploited ecosystems indicates that fishing also induced a decrease in the consumer biomass and a higher use of ecosystem production (increase of mean Ecotrophic Efficiency). The reduction in total biomass of the community confirms work by Worm et al. [93] who found an 11% decline in 144 stock assessment biomass time series since 1977, specifically in pelagic and demersal species in the North Atlantic. In addition, fishing uses the surplus production to some extent, and therefore will increase the explained mortality of the ecosystems. The lower primary production to total production ratio in fished ecosystems could result from fisheries targeting ecosystems with higher secondary production.

Among fishing indicators, we found that some ecosystem traits (latitude, ocean basins, depth) influence trophic level of the catch, while both depth and size affect the loss in production index, thus suggesting the need to account for these confounding traits when evaluating such fishing indicators and using them as ecosystem indicators. Nevertheless, the analysis also showed that, with the exception of trophic level of the catch, fishing indicators were overall less impacted by model covariates (i.e. factors that describe the food webs) than the ecological indicators. These indicators may be of special interest for future food web studies if correction for covariance is not possible. In general, both ecosystem dynamics (such as predator-prey interactions) and external factors (such as fishing) will have an impact on ecosystems and the strength of these impacts depends on a variety of factors [47], [94]–[97]. However, other variations such as economic drivers would also have to be taken into consideration in future studies.

The search for key functional groups in the 105 food webs showed that keystone groups had higher trophic level and mainly affected food webs as predators (top-down), whereas structural functional groups were benthic primary producers, which affected food webs mainly, but not exclusively, through bottom-up effects. Given their high overall impact, modification of the biomass of these key groups through anthropogenic-induced changes may produce important changes in food webs, possibly impairing ecosystem structure and functioning. Reducing top-down impacts exerted through predation by removing or depleting keystone groups (for instance) can cause ecological effects such as the increase of their prey [98], lower predatory mortality for individuals affected by disease or deficiencies, and can result in the decreased transfer efficiency of the ecosystem. On the contrary, modification of structuring groups that exert large effects on food webs through a prevalence of bottom-up effects implies potentially large impacts on the higher trophic levels. Given the prey-predator basis of models analysed, it is very likely that non-predatory roles exerted by structuring species (such as protection, habitat building, interference with physical variables) are underestimated in this analysis and thus the ecological role of these species may be even larger.

Notably, while structuring species appear to be evenly distributed according to ecosystem traits, keystone groups were especially prevalent in estuarine systems and systems smaller than 10 km^2^ and less than 5 m in depth. As the Indian Ocean modelled areas were mainly shallow, small estuaries, a significant proportion of keystone groups are identified. However, these results may also be due to the Indian Ocean being historically less impacted by fishing [53] or the fact that mainly bays and estuarine models of the Indian Ocean were available.

These results suggest that coastal and shallow areas with high physical/chemical variability such as estuaries are likely to host a relatively higher proportion of keystone functional groups. Such a result might be important, if further confirmed, for supporting the protection and conservation of these ecosystems, as these groups are often directly implicated with key marine ecosystem services including biodiversity and marine resources [99].

Overall, the disproportionate impacts of keystones and structuring groups [59] implies disproportionate effects if their biomass is modified, thus recommending particular caution when contemplating human impacts. Results of key groups' analysis, therefore, further encourage the protection of estuarine environments, already on the priority list for protection under the Ramsar Convention [100] for the goods and services they provide, for their high ecological value and of importance when valuing ecosystem services [101].

We found a significantly higher proportion of keystone functional groups in lightly exploited ecosystems in comparison to more exploited areas, despite a consistently stable proportion of structuring species. The lack of significant changes in the proportion of keystones in no-fishing models might be due to the low number of unexploited webs available for the analysis. Nevertheless, the significant result for the lightly fished ecosystems may indicate that fishing negatively affects keystones and/or that the keystone role is more prominent and distributed among functional groups in protected environments. The larger abundance of keystone functional groups in lightly exploited ecosystems confirmed previous results for the Mediterranean Sea [57] and highlights a possible effect of fishing in levelling out the species effects. We could not distinguish if the lower proportion of keystones in heavily exploited ecosystems was due to removal of these groups or if the keystone role was hampered by fishing, but we conclude that keystoneness is more clearly pronounced when fisheries exploitation is low. This insightful result merits further study to verify its generality. Overall the keystone groups appear to be more sensitive than structural species to ecosystem properties and exploitation. While their sensitivity might be due to their lower abundance, the large effects they produce in ecosystems through food web interactions make them optimal groups for signalling ecosystem disturbances and impairment, thus being good candidates for ecological indicators.

## Conclusions

Our results provide additional knowledge on how marine ecosystems structure and function, and the fact that different patterns occur in different ecosystems pose additional scientific questions and management challenges. For example, significant changes of food web indicators from marine ecosystems highlight the need to set well defined reference levels and thresholds when managing marine resources. It is not possible to set one reference level for all systems regardless of size, depth, or type of ecosystems. Nor it is useful to set reference levels for similar systems in different ocean basins, even if these systems seem to be similar in physical characteristics, i.e. being eastern boundary current systems. Since different baseline references exist and marine ecosystems seem to have intrinsic differences due to ecosystem dynamics, establishing absolute reference values for ecosystem indicators as a whole seems not to be a suitable solution to advance the ecosystem-based and precautionary approach. Reference levels for ecosystem indicators should be developed for individual ecosystems or ecosystems with the same typologies (similar location, ecosystem type, etc.) and not benchmarked against all other ecosystems.

## Supporting Information

Table S1 **Models used in this analysis with ecosystem descriptors and analyses performed (ENA  =  ecological network analysis, EwE  =  Ecopath and Ecosim).**
(DOCX)

File S1 **Description of Ecopath with Ecosim.**
(DOCX)

## References

[1] McCann K (2007) Protecting biostructure. Nature 446: 29. 17330028 10.1038/446029a

[2] Jackson JBC, Kirby MX, Berger WH, Bjorndal KA, Botsford LW, et al (2001) Historical Overfishing and the Recent Collapse of Coastal Ecosystems. Science 293: 629–638. 11474098 10.1126/science.1059199

[3] Hilborn R, Walters CJ (1992) Quantitative Fisheries Stock Assessment: Choice, Dynamics and Uncertainty. Boston: Kluwer Academic Publishers. 570 p.

[4] Christensen V, Guénette S, Heymans JJ, Walters C, Watson R, et al (2003) Hundred-year decline of North Atlantic predatory fishes. Fish and Fisheries 4: 1–24.

[5] Pikitch EK, Santora C, Babcock EA, Bakun A, Bonfil R, et al (2004) Ecosystem-Based Fishery Management. Science 305: 346–347. 15256658 10.1126/science.1098222

[6] Christensen V, Walters CJ (2004) Trade-offs in Ecosystem-scale Optimization of Fisheries Management Policies. Bulletin of Marine Science 74: 549–562.

[7] Heymans JJ, Sumaila UR, Christensen V (2009) Policy options for the northern Benguela ecosystem using a multispecies, multifleet ecosystem model. Progress in Oceanography 83: 417–425.

[8] Link J (2010) Ecosystem based fisheries management: Confronting Tradeofs. New York: Cambridge University Press.

[9] Christensen V, Walters CJ (2005) Using ecosystem modeling for fisheries management: Where are we? ICES CM 2005: 1–17.

[10] Heithaus MR, Frid A, Wirsing AJ, Worm B (2008) Predicting ecological consequences of marine top predator declines. Trends in Ecology and Evolution 23: 202–210. 18308421 10.1016/j.tree.2008.01.003

[11] Ulanowicz RE, Wulff F (1991) Comparing Ecosystem Structures: The Chesapeake Bay and the Baltic Sea. In: Cole J, Lovett G, Findlay S, editors. Comparative Analyses of Ecosystems: Patterns, Mechanisms, and Theories. 1 ed. New York: Springer-Verlag. pp. 140–166.

[12] Dunne JA (2006) The network structure of foodwebs. In: Pascual M, Dunne JA, editors. Ecological networks: linking structure to dynamics in food webs. Oxford: Oxford University Press. pp. 27–86.

[13] Perry RI, Cury P, Brander K, Jennings S, Möllmann C, et al (2010) Sensitivity of marine systems to climate and fishing: Concepts, issues and management responses. Contributions from Advances in Marine Ecosystem Modelling Research II 23-26 June 2008, Plymouth, UK 79: 427–435.

[14] Walters CJ, Martell SJD (2004) Fisheries ecology and management. Princeton: Princeton University Press. 399 p.

[15] Allesina S, Pascual M (2008) Network structure, predator–prey modules, and stability in large food webs. Theoretical Ecology 1: 55–64.

[16] Belgrano A, Scharler UM, Dunne J, Ulanowicz RE (2005) Aquatic food webs: an ecosystem approach. Oxford: Oxford University Press.

[17] Pinnegar JK, Blanchard JL, Mackinson S, Scott RD, Duplisea DE (2005) Aggregation and removal of weak-links in food-web models: system stability and recovery from disturbance. Ecological Modelling 184: 229–248.

[18] De Boer RJ (2012) Which of Our Modeling Predictions Are Robust? PLoS Comput Biol 8: e1002593. 22844235 10.1371/journal.pcbi.1002593 PMC3405990

[19] Shin YJ, Bundy A, Shannon LJ, Blanchard JL, Chuenpagdee R, et al (2012) Global in scope and regionally rich: an IndiSeas workshop helps shape the future of marine ecosystem indicators. Reviews in Fish Biology and Fisheries DOI 10.1007/s11160-012-9252-z: 1–11.

[20] Cury PM, Shannon LJ, Roux J-P, Daskalov GM, Jarre A, et al (2005) Trophodynamic indicators for an ecosystem approach to fisheries. ICES Journal of Marine Science 62: 430–442.

[21] Farnsworth KD, Lyashevska O, Fung T (2012) Functional complexity: The source of value in biodiversity. Ecological Complexity 11: 46–52.

[22] Christensen V, Pauly D (1993) Trophic models of aquatic ecosystems. ICLARM Conference Proceedings 26. Manila, Philippines: International Center for Living Resources Management. 1–390 p.

[23] Pauly D, Christensen V, Walters C (2000) Ecopath, Ecosim, and Ecospace as tools for evaluating ecosystem impacts of fisheries. ICES Journal of Marine Science 57: 697–706.

[24] Christensen V, Walters CJ (2004) Ecopath with Ecosim: methods, capabilities and limitations. Ecological Modelling 172: 109–139.

[25] Polovina JJ (1984) Model of a coral reef ecosystem I. The ECOPATH model and its application to French Frigate Shoals. Coral Reefs 3: 1–11.

[26] Christensen V, Pauly D (1992) ECOPATH II - a software for balancing steady-state ecosystem models and calculating network characteristics. Ecological Modelling 61: 169–185.

[27] Walters C, Pauly D, Christensen V, Kitchell JF (2000) Representing Density Dependent Consequences of Life History Strategies in Aquatic Ecosystems: EcoSim II. Ecosystems 3: 70–83.

[28] Christensen V, Walters C, Pauly D (2005) Ecopath with Ecosim: A User's guide. Vancouver, BC: Fisheries Centre, University of British Columbia. 154pp. p.

[29] Walters C, Christensen V, Pauly D (1997) Structuring dynamic models of exploited ecosystems from trophic mass-balance assessments. Reviews in Fish Biology and Fisheries 7: 139–172.

[30] Cohen JE, Luczak T, Newman CM, Zhou Z-M (1990) Stochastic structure and nonlinear dynamics of food webs: qualitative stability in a Lotka-Volterra Cascade Model. Proceedings of the Royal Society B: Biological Sciences 240: 607–627.

[31] Williams RJ, Martinez ND (2000) Simple rules yield complex food webs. Nature 404: 180–183. 10724169 10.1038/35004572

[32] Coll M, Lotze HK, Romanuk TN (2008) Structural degradation in Mediterranean Sea food webs: Testing ecological hypotheses using stochastic and mass-balance modelling. Ecosystems 11: 939–960.

[33] Lindeman RL (1942) The trophic-dynamic aspect of ecology. Ecology 23: 399–418.

[34] Odum EP (1969) The strategy of ecosystem development. Science 164: 262–270. 5776636 10.1126/science.164.3877.262

[35] Ulanowicz RE (1980) An hypothesis on the development of natural communities. Journal of theoretical Biology 85: 223–245. 7431954 10.1016/0022-5193(80)90019-3

[36] Ulanowicz RE (1983) Identifying the structure of cycling in ecosystems. Mathematical BioScience 65: 219–237.

[37] Moreno T, Castro JJ (1998) Trophic structure of the Maspalomas lagoon (Gran Canaria, Canary Islands), a regenerated ecosystem of brackish water. Boletin do Museu Municipal do Funchal (História Natural) Sup. no.5 245–261.

[38] Mackinson S, Daskalov G (2007) An ecosystem model of the North Sea for use in research supporting the ecosystem approach to fisheries management: description and parameterisation. Lowestoft: CEFAS. Cefas Science Series Technical Report 142 Cefas Science Series Technical Report 142. 200pp. p.

[39] Libralato S, Pastres R, Pranovi F, Raicevich S, Granzotto A, et al (2002) Comparison between the energy flow networks of two habitats in the Venice Lagoon. Marine Ecology 23: 228–236.

[40] Stobberup KA, Ramos VDM, Coelho ML (2004) Ecopath model of the Cape Verde coastal ecosystem. In: Palomares ML, Pauly D, editors. West African marine ecosystems: models and fisheries impacts: Fisheries Center Research Reports 12(7). Vancouver, BC: UBC Fisheries Centre. pp. 39–56.

[41] Heymans JJ, Howell KL, Ayers M, Burrows MT, Gordon JDM, et al (2011) Do we have enough information to apply the ecosystem approach to management of deep-sea fisheries? An example from the West of Scotland. ICES Journal of Marine Science 68: 265–280.

[42] Pinnegar JK, Polunin NVC (2004) Predicting indirect effects of fishing in Mediterranean rocky littoral communities using a dynamic simulation model. Ecological Modelling 172: 249–267.

[43] Dunne JA, Williams RJ, Martinez ND (2002) Food-web structure and network theory: The role of connectance and size. Proceedings of the National Academy of Sciences of the United States of America 99: 12917–12922. 12235364 10.1073/pnas.192407699 PMC130560

[44] Finn JT (1976) Measures of ecosystem structure and function derived from analysis of flows. Journal of theoretical Biology 56: 363–380. 944838 10.1016/s0022-5193(76)80080-x

[45] Ulanowicz RE (1986) Growth and Development: Ecosystems Phenomenology. Lincoln, NE: toExcel Press. 203 p.

[46] Ulanowicz RE (2000) Toward the Measurement of Ecological Integrity. In: Pimentel D, Westra L, Noss RF, editors. Ecological integrity: integrating environment, conservation, and health. Washington DC: Island Press. pp. 99–113.

[47] Heymans JJ, Guénette S, Christensen V (2007) Evaluating network analysis indicators of ecosystem status in the Gulf of Alaska. Ecosystems 10: 488–502.

[48] Ulanowicz RE (2004) Quantitative methods for ecological network analysis. Computational Biology and Chemistry 28: 321–339. 15556474 10.1016/j.compbiolchem.2004.09.001

[49] Libralato S (2008) System Omnivory Index. In: Jørgensen SE, Fath BD, editors. Ecological Indicators Oxford: Elsevier. pp. 3472–3477.

[50] Pauly D, Christensen V, Dalsgaard J, Froese R, Torres FJ (1998) Fishing down marine food webs. Science 279: 860–863. 9452385 10.1126/science.279.5352.860

[51] Libralato S, Coll M, Tudela S, Palomera I, Pranovi F (2008) Novel index for quantification of ecosystem effects of fishing as removal of secondary production. Marine Ecology Progress Series 355: 107–129.

[52] Libralato S, Christensen V, Pauly D (2006) A method for identifying keystone species in food web models. Ecological Modelling 195: 153–171.

[53] Coll M, Libralato S, Tudela S, Palomera I, Pranovi F (2008) Ecosystem Overfishing in the Ocean. PLoS ONE 3: e3881. 19066624 10.1371/journal.pone.0003881 PMC2587707

[54] Mora C, Myers RA, Coll M, Libralato S, Pitcher TJ, et al (2009) Management Effectiveness of the World's Marine Fisheries. PLoS Biol 7: e1000131. 19547743 10.1371/journal.pbio.1000131 PMC2690453

[55] Murawski SA (2000) Definitions of overfishing from an ecosystem perspective. ICES Journal of Marine Science 57: 649–658.

[56] Heymans JJ, Coll M, Libralato S, Christensen V (2012) 9.06 Ecopath theory, modelling and application to coastal ecosystems. In: Wolanski E, McLusky DS, editors. Treatise on Estuarine and Coastal Science: Elsevier. pp. 93–113.

[57] Coll M, Libralato S (2012) Contributions of food web modelling to the ecosystem approach to marine resource management in the Mediterranean Sea. Fish and Fisheries 13: 60–88.

[58] Anderson MA, Gorley RN, Clarke KR (2008) PERMANOVA+ for PRIMER: Guide to software and statistical methods. Plymouth, UK: PRIMER-E.

[59] Power ME, Tilman D, Estes JA, Menge BA, Bond WJ, et al (1996) Challenges in the Quest for Keystones. BioScience 46: 609–620.

[60] Piraino S, Fanelli G, Boero F (2002) Variability of species' roles in marine communities: change of paradigms for conservation priorities. Marine Biology 140: 1067–1074.

[61] Ulanowicz RE, Puccia CJ (1990) Mixed trophic impacts in ecosystems. Coenoses 5(1): 7–16.

[62] Greenacre MJ (1984) Theory and applications of corespondence analysis. London: Academic Press.

[63] Kakarenkov V, Legendre P (2002) Nonlinean redundancy analysis and cononical correspondence analysis based on polynomial regression. Ecology 83: 1146–1161.

[64] Fellenberg K, Hauser NC, Brors B, Neutzner A, Hoheisel JD, et al (2001) Correspondence analysis applied to microarray data. Proceedings of the National Academy of Sciences 98: 10781–10786. 10.1073/pnas.181597298 PMC58552 11535808

[65] Lotze HK, Lenihan HS, Bourque BJ, Bradbury RH, Cooke RG, et al (2006) Depletion, Degradation, and Recovery Potential of Estuaries and Coastal Seas. Science 312: 1806–1809. 16794081 10.1126/science.1128035

[66] Costello MJ, Coll M, Danovaro R, Halpin P, Ojaveer H, et al (2010) A Census of Marine Biodiversity Knowledge, Resources, and Future Challenges. PLoS ONE 5: e12110. 20689850 10.1371/journal.pone.0012110 PMC2914025

[67] Plagányi ÉE, Butterworth DS (2004) A critical look at the potential of Ecopath with Ecosim to assist in practical fisheries management. African Journal of Marine Science 26: 261–287.

[68] Plagányi ÉE (2007) Models for an ecosystem approach to fisheries. FAO Fisheries Technical Paper No. 477. Rome: FAO. 477 477. 108 p.

[69] Dame JK, Christian RR (2007) A statistical test of network analysis: Can it detect differences in food web properties? Ecosystems 10: 906–923.

[70] Fulton EA, Smith ADM, Punt AE (2005) Which ecological indicators can robustly detect effects of fishing? ICES J Mar Sci 62: 540–551.

[71] Lassen H, Pedersen SA, Frost H, Hoff A (2013) Fishery management advice with ecosystem considerations. ICES Journal of Marine Science: Journal du Conseil 70: 471–479.

[72] EC (2008) Directive 2008/56/EC, of 17 June 2008 establishing a framework for community action in the field of marine environmental policy (Marine Strategy Framework Directive). 22.p.

[73] Shin YJ, Bundy A, Shannon LJ, Simier M, Coll M, et al (2010) Can simple be useful and reliable? Using ecological indicators to represent and compare the states of marine ecosystems. ICES J Mar Sci 67: 717–731.

[74] Samhouri JF, Levin PS, Ainsworth CH (2010) Identifying Thresholds for Ecosystem-Based Management. PLoS ONE 5: e8907. 20126647 10.1371/journal.pone.0008907 PMC2811186

[75] Levin PS, Fogarty MJ, Murawski SA, Fluharty D (2009) Integrated Ecosystem Assessments: Developing the Scientific Basis for Ecosystem-Based Management of the Ocean. PLoS Biol 7: e1000014. 10.1371/journal.pbio.1000014 PMC2628402 19166267

[76] Gribble NA (2003) GBR-prawn: modelling ecosystem impacts of changes in fisheries management of the commercial prawn (shrimp) trawl fishery in the far northern Great Barrier Reef. Fisheries Research 65: 493–506.

[77] Bozec Y-M, Gascuel D, Kulbicki M (2004) Trophic model of lagoonal communities in a large open atoll (Uvea, Loyalty islands, New Caledonia). Aquatic Living Resources 17: 151–162.

[78] Venier JM, Pauly D (1997) Trophic dynamics of a Florida Keys coral reef ecosystem. 8th International Coral Reef Symposium. Panama City: Smithsonian Tropica Research Institute, Balboa, Panama. pp. 915–920.

[79] Opitz S (1993) A quantitative model of the trophic interactions in a Caribbean coral reef ecosystem. In: Christensen V, Pauly D, editors. Trophic models of aquatic ecosystems ICLARM Conference Proceedings 26. 1 ed. Manila, Philippines: International Center for Living Aquatic Resources Management. pp. 259–267.

[80] Buzzelli C (2012) 9.13 Ecosystem modelling in small subtropical estuaries and embayments. In: Wolanski E, McLusky DS, editors. Treatise on Estuarine and Coastal Science: Elsevier.pp. 331–353.

[81] Baird D, Ulanowicz RE (1993) Comparative study on the trophic structure, cycling and ecosystem properties of four tidal estuaries. Marine Ecology Progress Series 99: 221–237.

[82] Baird D, McGlade JM, Ulanowicz RE (1991) The comparative ecology of six marine ecosystems. Philosophical Transactions of the Royal Society of London, B 333: 15–29.

[83] Jarre-Teichmann A, Christensen V (1998) Comparative modelling of trophic flows in four large upwelling ecosystems: global vs. local effects. In: Cury P, Mendelssohn R, Roy C, Bakun A, Durand MH, et al.., editors. Global vs local changes in upwelling ecosystems Proceedings of the first CEOS symposium, 5–9 September 1994 Monterey, CA.

[84] Jarre-Teichmann A, Shannon LJ, Moloney CL, Wickens PA (1998) Comparing trophic flows in the southern Benguela to those in other upwelling ecosystems. South African Journal of marine Science 19: 391–414.

[85] Heymans JJ, Baird D (2000) A carbon flow model and network analysis of the northern Benguela upwelling system, Namibia. Ecological Modelling 126: 9–32.

[86] Vasconcellos M, Mackinson S, Sloman K, Pauly D (1997) The stability of trophic mass-balance models of marine ecosystems: a comparative analysis. Ecological Modelling 100: 125–134.

[87] Heymans JJ, Baird D (2000) Network analysis of the northern Benguela ecosystem by means of NETWRK and ECOPATH. Ecological Modelling 131: 97–119.

[88] Helmke P, Neuer S, Lomas MW, Conte M, Freudenthal T (2010) Cross-basin differences in particulate organic carbon export and flux attenuation in the subtropical North Atlantic gyre. Deep Sea Research Part I: Oceanographic Research Papers 57: 213–227.

[89] Carr M-E, Kearns EJ (2003) Production regimes in four Eastern Boundary Current systems. Deep-Sea Research II 50: 3199–3221.

[90] Tomczak MT, Müller-Karulis B, Järv L, Kotta J, Martin G, et al (2009) Analysis of trophic networks and carbon flows in south-eastern Baltic coastal ecosystems. Progress in Oceanography 81: 111–131.

[91] Lotze HK, Coll M, Dunne JA (2011) Historical Changes in Marine Resources, Food-web Structure and Ecosystem Functioning in the Adriatic Sea, Mediterranean. Ecosystems 14: 198–222.

[92] Tomczak MT, Heymans JJ, Yletyinen J, Niiranen S, Otto SA, et al (2013) Ecological Network Indicators of Ecosystem Status and Change in the Baltic Sea. PLoS ONE 8: e75439. 24116045 10.1371/journal.pone.0075439 PMC3792121

[93] Worm B, Hilborn R, Baum JK, Branch TA, Collie JS, et al (2009) Rebuilding Global Fisheries. Science 325: 578–585. 19644114 10.1126/science.1173146

[94] Shannon LJ, Christensen V, Walters CJ (2004) Modelling stock dynamics in the southern Benguela ecosystem for the period 1978–2002. African Journal of Marine Science 26: 179–196.

[95] Heymans JJ, Shannon LJ, Jarre A (2004) Changes in the northern Benguela ecosystem over three decades: 1970s, 1980s and 1990s. Ecological Modelling 172: 175–195.

[96] Heymans JJ, Guénette S, Christensen V, Trites A (2005) Changes in the Gulf of Alaska ecosystems due to ocean climate change and fishing. ICES CM 2005: 1–31.

[97] Heymans JJ (2004) The effects of internal and external control on the northern Benguela ecosystem. In: Sumaila UR, Steinshamn SI, Skog M, Boyer DC, editors. Ecological, economic and social aspects of Namibian fisheries. Netherlands: Eburon. pp. 29–52.

[98] Paine RT (1969) A note on trophic complexity and community stability. American Naturalist 103: 91–93.

[99] Butchart SHM, Walpole M, Collen B, van Strien A, Scharlemann JPW, et al (2010) Global Biodiversity: Indicators of recent declines. Science Express 29 April 2010 1–9. 10.1126/science.1187512 20430971

[100] UN (1987) Convention on Wetlands of International Importance especially as Waterfowl Habitat. Ramsar (Iran), 2 February 1971. UN Treaty Series No. 14583. As amended by the Paris Protocol, 3 December 1982, and Regina Amendments, 28 May 1987.

[101] Costanza R, d'Arge R, de Groot R, Farber S, Grasso M, et al (1997) The value of the world's ecosystem services and natural capital. Nature 387: 253–260.

[102] Pranovi F, Libralato S, Raicevich S, Granzotto A, Pastres R, et al (2003) Mechanical clam dredging in Venice lagoon: ecosystem effects evaluated with a trophic mass-balance model. Marine Biology 143: 393–403.

[103] Essington TE (2007) Evaluating the sensitivity of a trophic mass-balance model (Ecopath) to imprecise data inputs. Canadian Journal of Fisheries and Aquatic Science 64: 628–637.

