# Supplementary material for: Global Patterns in Ecological Indicators of Marine Food Webs: A Modelling Approach
Source: PLoS One. 2014 Apr 24;9(4):e95845. doi: 10.1371/journal.pone.0095845 (PMC3998982; doi:10.1371/journal.pone.0095845)
Supplement: Table S1 — Models used in this analysis with ecosystem descriptors and analyses performed (ENA = ecological network analysis, EwE = Ecopath and Ecosim). (DOCX) [file pone.0095845.s001.docx]

### Supporting Information

**Table S1:** Models used in this analysis with ecosystem descriptors and analyses performed (ENA = ecological network analysis, EwE = Ecopath and Ecosim).

| **Area** | **No** | **Country** | **Model** | **Size (km^2^)** | **Ecosystem type** | **Exploitation** | **Analyses performed*** | **Groups** | **Depth (m)** | **Date** | **Latitude** | **Longitude** | **References** |
| --- | --- | --- | --- | --- | --- | --- | --- | --- | --- | --- | --- | --- | --- |
| Africa | 1 | Cape Verde | Cape Verde | 789,400 | Upper slope | yes | Ecopath | 31 | 200-3000 | 1981-1985 | 12.8-19N | 27-20 W | [[1](#_ENREF_1)] |
|  | 2 | Gambia | Gambia | 10,500 | Shelf | yes | Ecopath | 23 | 0-200 | 1995 | 13.04-13.6N | 20.3-162W | [[2](#_ENREF_2)] |
|  | 3 | Ghana | Sakumo Lagoon | 1 | Lagoon | yes | Ecopath, Ecospace | 14 | <10 | Early 1970s | 5 N | 2 W | [[3](#_ENREF_3)] |
|  | 4 | Guinea | Guinea | 43,000 | Shelf | yes | Ecopath | 44 | 0-200 | 1998 | 9.03-10.50N | 13.19-13.17W | [[4](#_ENREF_4)] |
|  | 5 | Guinea-Bissau | Guinea-Bissau | 10,500 | Shelf | yes | Ecopath | 32 | 200 | 1990-1992 | 11-12.30N | 17-15.5W | [[5](#_ENREF_5)] |
|  | 6 | Mauritania | Mauritania | 230,000 | Shelf | yes | Ecopath | 38 | 0-200 | 1998 | 16.04-20.50N | 23-16W | [[6](#_ENREF_6)] |
|  | 7 | Morocco | Morocco | 586,900 | Shelf | yes | Ecopath | 38 | 0-200 | 1985-1986 | 36-20 N | 20-6W | [[7](#_ENREF_7)] |
|  | 8 | Mozambique | Maputo | 1,100 | Bay | yes | Ecopath | 10 | 15 | 1970-80s | 32.42 S | 26 E | [[8](#_ENREF_8)] |
|  | 9 | Namibia | Northern Benguela | 179,000 | Shelf | yes | EwE fitting, policy | 32 | 500 | 1956 | 15-29 S | 10-17 E | [[9](#_ENREF_9)] |
|  | 10 | Senegal | Senegambien | 27,600 | Shelf | yes | Ecopath | 18 | 0-200 | 1990s | 12.5-16.5N | 20-16.5W | [[10](#_ENREF_10)] |
|  | 11 | Sierra Leone | Sierre Leone 1964 | 27,500 | Shelf | yes | Ecopath | 43 | 200 | 1964 | 7-10 N | 11-16 W | [[11](#_ENREF_11)] |
|  | 12 | Sierra Leone | Sierre Leone 1978 | 27,500 | Shelf | yes | Ecopath | 43 | 200 | 1978 | 7-10 N | 11-16 W | [[11](#_ENREF_11)] |
|  | 13 | Sierra Leone | Sierre Leone 1990 | 27,500 | Shelf | yes | Ecopath | 43 | 200 | 1990 | 7-10 N | 11-16 W | [[11](#_ENREF_11)] |
|  | 14 | South Africa | Gamtoos Estuary | 1 | Estuary | recreational fishing only | EwE, ENA | 33 | 3.5 | 1980-2000s | 34 S | 25 E | [[12](#_ENREF_12)] |
|  | 15 | South Africa | Sundays Estuary | 2 | Estuary | recreational fishing only | EwE, ENA | 33 | 3 | 1980-2000s | 34 S | 25 E | [[12](#_ENREF_12)] |
|  | 16 | South Africa | Kromme Estuary | 3 | Estuary | recreational fishing only | EwE, ENA | 33 | 2.8 | 1980-2000s | 34 S | 24 E | [[12](#_ENREF_12)] |
|  | 17 | South Africa | Swartkops Estuary | 5 | Estuary | yes | EwE, ENA | 33 | 3 | 1980-2000s | 34 S | 26 E | [[12](#_ENREF_12)] |
| Australasia | 18 | Australia | Great Barrier Reef-prawn | 10,000 | Reef | yes | EwE | 25 | 50 | 1997 | 10-12 S | 142-145 E | [[13](#_ENREF_13)] |
|  | 19 | Bangladesh | Bay of Bengal | 166,000 | Bay | yes | Ecopath, ENA | 15 | 150 | 1984-1986 | 20-22 N | 90-92 E | [[14](#_ENREF_14)] |
|  | 20 | Brunei Darussalam | Brunei | 7,396 | Coastal | yes | Ecopath | 13 | 100 | 1989 | 4-5 N | 114-115 E | [[15](#_ENREF_15)] |
|  | 21 | China | East China Sea | 770,000 | Shelf | yes | Ecopath, ENA | 45 | 370 | 1997-2000 | 23-34 N | 122-128 E | [[16](#_ENREF_16)] |
|  | 22 | India | SE Arabian Sea | 27,000 | Coastal | yes | Ecopath | 24 | 200 | 1999-2001 | 11-18 N | 74-78E | [[17](#_ENREF_17)] |
|  | 23 | India | Southwest coast of India | 75,390 | Shelf | yes | Ecopath, ENA | 11 | 50 | 1994-1996 | 8-16 N | 72-77 E | [[18](#_ENREF_18)] |
|  | 24 | Malaysia | West coast of Sabah | 28,000 | Shelf | yes | Ecopath, ENA | 29 | 60 | 1972 | 5-8 N | 112-117 E | [[19](#_ENREF_19)] |
|  | 25 | Malaysia | West coast of Sarawak | 125,000 | Shelf | yes | Ecopath, ENA | 29 | 60 | 1972 | 1-7 N | 109-114 E | [[19](#_ENREF_19)] |
|  | 26 | New Caledonia | Loyalty Islands Atoll | 872 | Reef | no | Ecopath | 25 | 20 | 1980s | 20 S | 116 E | [[20](#_ENREF_20)] |
|  | 27 | Philippines | San Pedro Bay, Leyte | 625 | Bay | yes | Ecopath, ENA | 16 | 20 | 1994-1995 | 25 N | 11 E | [[21](#_ENREF_21)] |
|  | 28 | Philippines | San Miguel Bay | 1,115 | Bay | yes | Ecopath, ENA | 19 | 20 | 1992-1994 | 13-14 N | 123 E | [[22](#_ENREF_22)] |
|  | 29 | Taiwan | Kuosheng Bay | 8 | Bay | yes | Ecopath, ENA | 17 | 15 | 1998-2003 | 25 N | 121 E | [[23](#_ENREF_23)] |
|  | 30 | Taiwan | Lagoon Chiku - Taiwan | 10 | Lagoon | yes | Ecopath, ENA | 13 | 10 | 1997 | 23 N | 120 E | [[24](#_ENREF_24)] |
|  | 31 | Thailand | Gulf of Thailand | 304,000 | Coastal | yes | EwE, Policy | 40 | 58 | 1973 | 6-13 N | 99-104 E | [[25](#_ENREF_25)], [[26](#_ENREF_26)] |
| Europe | 32 | Canary Islands | Maspalomas Lagoon | 0.05 | Lagoon | no | Ecopath, ENA | 6 | 1.5 | 1993-1994 | 27 N | 15 W | [[27](#_ENREF_27)] |
|  | 33 | Estonia, Latvia | Gulf of Riga | 240 | Coastal | yes | Ecopath, ENA | 12 | 5 | 1990-2000s | 57 N | 24 E | [[28](#_ENREF_28)] |
|  | 34 | Estonia, Latvia | Parnu Bay | 700 | Bay | yes | Ecopath, ENA | 12 | 23 | 1990-2000s | 58 N | 24 E | [[28](#_ENREF_28)] |
|  | 35 | France | Bay of Calvi | 22 | Bay | low | EwE | 27 | 18 | 1998 | 42 N | 8 E | [[29](#_ENREF_29)] |
|  | 36 | France | Bay of Somme | 50 | Bay | no | Ecopath, ENA | 9 | 11 | 1990s | 50 N | 1 E | [[30](#_ENREF_30)] |
|  | 37 | France | Seine Estuary | 81 | Estuary | no | Ecopath, ENA | 15 | 5 | 1996-1999 | 49 N | 0.1 W | [[31](#_ENREF_31)] |
|  | 38 | France | Etang de Thau | 88 | Lagoon | yes | Ecopath | 11 | 10 | 1980s | 43.3 N | 3.3 E | [[32](#_ENREF_32)] |
|  | 39 | France | Gironde Estuary | 625 | Estuary | yes | Ecopath, ENA | 18 | 20 | 1980s-1990s | 45 N | 1 E | [[33](#_ENREF_33)] |
|  | 40 | Greece | Aegean model | 8,374 | Upper slope | yes | Ecopath | 40 | 20-300 | 2003-2006 | 40-41N | 24-26 E | [[34](#_ENREF_34)] |
|  | 41 | Iceland | Iceland | 115,000 | Shelf | yes | EwE | 21 | 200 | 1997 | 63-67 N | 12-26 W | [[35](#_ENREF_35)] |
|  | 42 | Iceland | Iceland - 1950 | 376,766 | Shelf | yes | EwE, fitting | 24 | 200 | 1950 | 63-67 N | 10-25 W | [[36](#_ENREF_36)] |
|  | 43 | Italy | Orbetello 1995 | 25 | Lagoon | yes | Ecopath, ENA | 12 | 1 | 1995 | 42 N | 11 E | [[37](#_ENREF_37)] |
|  | 44 | Italy | Orbetello Lagoon | 25 | Lagoon | yes | Ecopath, ENA | 12 | 1 | 1996 | 42 N | 11 E | [[37](#_ENREF_37)] |
|  | 45 | Italy | Lagoon of Venice | 35 | Lagoon | yes | Ecopath, ENA | 16 | 0.5 | 1990s | 45 N | 12 E | [[38](#_ENREF_38)] |
|  | 46 | Italy | Venice Lagoon (Seagrass habitat) | 40 | Lagoon | yes | Ecopath, ENA | 21 | 0.5 | 1990s | 45 N | 12 E | [[39](#_ENREF_39)] |
|  | 47 | Italy | Miramare Natural Marine Reserve | 120 | Coastal | no | Ecopath, ENA | 18 | 18 | 1986 | 45 N | 13 E | [[40](#_ENREF_40)] |
|  | 48 | Italy | Venice Lagoon (Tapes habitat) | 134 | Lagoon | yes | Ecopath, ENA | 27 | 0.5 | 1990s | 45 N | 12 E | [[41](#_ENREF_41)] |
|  | 49 | Italy | North-Central Adriatic 1970s | 55,500 | Shelf | yes | EwE, fitting | 40 | 10-200 | 1975-1980 | 42-46 N | 12-17 E | [[42](#_ENREF_42)] |
|  | 50 | Italy | North-Central Adriatic 1990s | 55,500 | Shelf | yes | Ecopath, ENA | 40 | 10-200 | mid 1990 | 42-46 N | 12-16 E | [[43](#_ENREF_43)] |
|  | 51 | Lituania | Lithuanian Coast | 7,000 | Coastal | yes | Ecopath, ENA | 12 | 14 | 1990-2000s | 56 N | 21 E | [[28](#_ENREF_28)] |
|  | 52 | Norway | Sorfjord | 55 | Bay | low | Ecopath | 27 | 125 | 1990s | 69,35 N | 19,45 E | [[44](#_ENREF_44)] |
|  | 53 | Poland, Lituania | Puck Bay | 359 | Bay | yes | Ecopath, ENA | 12 | 8.5 | 1990-2000s | 54.5 N | 19.5 E | [[28](#_ENREF_28)] |
|  | 54 | Poland, Lituania | Curonian Lagoon | 1,584 | Lagoon | yes | Ecopath, ENA | 12 | 3.5 | 1990-2000s | 55.5 N | 21 E | [[28](#_ENREF_28)] |
|  | 55 | Spain | Catalan sea 1980s | 4,500 | Upper slope | yes | EwE, ENA | 40 | 50-400 | 1976-1980 | 40-41N | 0-1E | [[45](#_ENREF_45)] |
|  | 56 | Spain | Catalan sea 1990s | 4,500 | Upper slope | yes | Ecopath and Ecosim, ENA | 40 | 50-400 | 1990-2000 | 40-41N | 0-1E | [[46](#_ENREF_46)] |
|  | 57 | Spain | Catalan sea 2003 | 4,500 | Upper slope | yes | EwE, ENA | 40 | 50-400 | 2003 | 40-41N | 0-1E | [[47](#_ENREF_47)] |
|  | 58 | Spain | Cantabrian Sea | 16,000 | Coastal | yes | Ecopath, ENA | 28 | 200 | 1994 | 43-45 N | 2-8 W | [[48](#_ENREF_48)] |
|  | 59 | Sweden, Finland, Poland | Baltic Sea (NEST) | 211,069 | Shelf | yes | EwE, fitting | 16 | <100 | 1974 | 54-60 N | 12-29 W | [[49](#_ENREF_49)], [[50](#_ENREF_50)] |
|  | 60 | Sweden, Finland, Poland | Baltic Sea | 240,000 | Shelf | yes | EwE, fitting | 16 | <100 | 1974 | 54-60 N | 12-29 W | [[51](#_ENREF_51)] |
|  | 61 | UK | West Coast of Scotland | 31,085 | Shelf | yes | EwE, Ecospace | 37 | 200 | 1995-2000 | 55-57 N | 5-8 W | [[52](#_ENREF_52)], [[53](#_ENREF_53)] |
|  | 62 | UK | Western English Chanel | 56,452 | Shelf | yes | EwE, fitting | 52 | 100 | 1973 | 48-50 N | 1-6 W | [[54](#_ENREF_54)], [[55](#_ENREF_55)] |
|  | 63 | UK | English Channel 1995 | 89,607 | Shelf | yes | Ecopath | 50 | 100 | 1995 | 49-50 N | 2 W - 2 E | [[56](#_ENREF_56)] |
|  | 64 | UK | North Sea 1880 | 570,000 | Shelf | yes | Ecopath | 46 | 200 | 1880 | 51-60 N | 2.5 W - 8.5 E | [[57](#_ENREF_57)] |
|  | 65 | UK | North Sea 1991 | 575,000 | Shelf | yes | EwE, fitting, Ecospace | 68 | 90 | 1991 | 51-62 N | 4W - 9 E | [[58](#_ENREF_58)] |
|  | 66 | UK | Deep sea West Coast of Scotland | 75,539 | Upper slope | yes | EwE, fitting | 39 | 400-2000 | 1974 | 54-60 N | 5-11 W | [[59](#_ENREF_59)], [[60](#_ENREF_60)] |
| North America | 67 | Canada | Strait of Georgia | 6,900 | Shelf | yes | Ecopath | 27 | ean 156, ax 420 | 1980s | 48-49 N | 122-123 W | [[61](#_ENREF_61)] |
|  | 68 | Canada | Eastern Scotian Shelf 1980s | 102,325 | Shelf | yes | Ecopath | 39 | 400 | mid-1980s | 42-45 N | 57-63 | [[62](#_ENREF_62)] |
|  | 69 | Canada | Eastern Scotian Shelf 1990s | 102,325 | Shelf | yes | Ecopath | 39 | 400 | mid-1990s | 42-45 N | 57-63 | [[62](#_ENREF_62)] |
|  | 70 | Canada | Northern Gulf of St. Lawrence 1980s | 103,812 | Shelf | yes | Ecopath | 32 | 480 | mid-1980s | 48-51 N | 66-57 | [[63](#_ENREF_63)], [[64](#_ENREF_64)] |
|  | 71 | Canada | Northern Gulf of St. Lawrence 1989s | 103,812 | Shelf | yes | Ecopath | 32 | 480 | mid-1990s | 48-51 N | 66-57 | [[63](#_ENREF_63)], [[64](#_ENREF_64)] |
|  | 72 | Canada | Northern Gulf of St. Lawrence 2000s | 103,812 | Shelf | yes | Ecopath | 32 | 480 | early-2000s | 48-51 N | 66-57 | [[63](#_ENREF_63)], [[64](#_ENREF_64)] |
|  | 73 | Canada | Southern Gulf of St. Lawrence 1980s | 103,812 | Shelf | yes | Ecopath | 30 | 480 | mid-1980s | 45-48 N | 59-65 | [[63](#_ENREF_63)], [[64](#_ENREF_64)] |
|  | 74 | Canada | Southern Gulf of St. Lawrence 1990s | 103,812 | Shelf | yes | Ecopath | 30 | 480 | mid-1990s | 45-48 N | 59-65 | [[63](#_ENREF_63)], [[64](#_ENREF_64)] |
|  | 75 | Mexico | Tampamachoco Lagoon | 15 | Lagoon | yes | Ecopath | 23 | <10 | 1986 | 21 N | 97 W | [[65](#_ENREF_65)] |
|  | 76 | Mexico | Celestun Lagoon | 28 | Lagoon | yes | Ecopath | 19 | 3 | 1992-1994 | 20 N | 90 W | [[66](#_ENREF_66)], [[67](#_ENREF_67)] |
|  | 77 | Mexico | Huizache-Caimanero | 175 | Lagoon | yes | EwE, fitting, policy | 26 | 0.85 | 1984-1986 | 23 N | 106 W | [[68](#_ENREF_68)], [[69](#_ENREF_69)], [[70](#_ENREF_70)] |
|  | 78 | Mexico | Campeche Bank | 250 | Shelf | yes | Ecopath | 19 | <200 | 1985-1990 | 20 N | 90 W | [[71](#_ENREF_71)] |
|  | 79 | Mexico | Tamaihua Lagoon | 800 | Lagoon | yes | Ecopath | 13 | <10 | 1980s | 21.5 N | 97.5 W | [[72](#_ENREF_72)] |
|  | 80 | Mexico | Terminos Lagoon | 2,500 | Lagoon | yes | Ecopath, ENA | 20 | 3.5 | 1980s | 18 N | 91 W | [[73](#_ENREF_73)] |
|  | 81 | Mexico | Seagrass&Mangrove Terminos Lagoon | 3,670 | Lagoon | yes | Ecopath, ENA | 16 | 3.5 | early 1990s | 18-20 N | 91-92 W | [[74](#_ENREF_74)] |
|  | 82 | Mexico | Alto Golfo de California | 7,200 | Shelf | yes | Ecopath, ENA | 29 | 200 | 1980-2000 | 29-32 N | 112-115 W | [[75](#_ENREF_75)] |
|  | 83 | Mexico | Central Gulf of California | 27,900 | Shelf | yes | EwE, fitting, policy | 27 | >2000 | 1978-1979 | 25-29 N | 109-112 E | [[76](#_ENREF_76)] |
|  | 84 | Mexico | Northern Gulf of California | 36,000 | Shelf | yes | EwE, fitting | 34 | 400 | 1960 | 29-32 N | 112-114 W | [[77](#_ENREF_77)] |
|  | 85 | Mexico | Sonda de Campeche | 65,000 | Shelf | yes | Ecopath, ENA | 19 | <200 | 1980s | 20 N | 91-94 W | [[78](#_ENREF_78)] |
|  | 86 | Mexico | Yucatan Continental Shelf | 100,000 | Shelf | yes | Ecopath, ENA | 21 | 200 | 1980s | 19-24 N | 85-92 W | [[79](#_ENREF_79)] |
|  | 87 | Mexico | Gulf of Mexico | 500,000 | Shelf | yes | Ecopath | 15 | <200 | 1980s | 18-30 N | 82-97 W | [[80](#_ENREF_80)] |
|  | 88 | Mexico | La Paz Bay | 1,500 | Bay | yes | EwE, fitting, policy | 22 | <200 | 1990s | 24-25 N | 110-111 W | [[81](#_ENREF_81)], [[82](#_ENREF_82)] |
|  | 89 | Mexico | Mandinga Lagoon | 10 | Lagoon | no | Ecopath | 20 | 3 | 1982-1983 | 18 N | 94 W | [[83](#_ENREF_83)] |
|  | 90 | Mexico | Western Gulf of Mexico | 1,500,000 | Coastal | yes | Ecopath, ENA | 24 | 200 | 1970s | 18-22 N | 96-97 W | [[84](#_ENREF_84)] |
|  | 91 | USA | Prince William Sound 1980s | 8,800 | Shelf | yes | EwE | 19 | 800 | 1980-1989 | 61 N | 147-148 W | [[85](#_ENREF_85)] |
|  | 92 | USA | Prince William Sound 1990s | 8,800 | Shelf | yes | EwE, policy | 48 | 800 | 1994-1996 | 61 N | 147-148 W | [[86](#_ENREF_86)] |
|  | 93 | USA | Aleutians Islands | 56,936 | Shelf | yes | EwE, fitting | 40 | 500 | 1963 | 50-54 N | 170 W - 170 E | [[87](#_ENREF_87)] |
|  | 94 | USA | Western Bering Sea | 1,358,000 | Shelf | yes | Ecopath | 36 | >200 | 1980-1985 | 52-62 N | 160-180 E | [[88](#_ENREF_88)] |
|  | 95 | USA | Monterey Bay | 909 | Bay | yes | Ecopath | 16 | 1000 | 1980s | 36-37 N | 121-122 W | [[89](#_ENREF_89)] |
|  | 96 | USA | Looe Key Sanctuary | 30 | Reef | no | Ecopath | 20 | <50 | 1980s | 24 N | 81 W | [[90](#_ENREF_90)] |
|  | 97 | USA | West Florida Shelf | 170,000 | Shelf | yes | Ecopath | 59 | 200 | 1960-1990 | 24-30 N | 80-87 W | [[91](#_ENREF_91)] |
| South America | 98 | Brazil | Southern Brazil Shelf | 28,661 | Shelf | yes | EwE, fitting | 13 | 200 | 1950 | 32-43 S | 51-54 W | [[92](#_ENREF_92)] |
|  | 99 | Brazil | South Brazil Bight | 97,000 | Shelf | yes | EwE | 25 | 100 | 1998-1999 | 23-28 S | 42-48 W | [[93](#_ENREF_93)] |
|  | 100 | Chile | Tongoy Bay | 60 | Bay | yes | Ecopath, ENA | 17 | 90 | 1978-1989 | 30S | 71 W | [[94](#_ENREF_94)] |
|  | 101 | Chile | Central Chile | 50,042 | Shelf | yes | Ecopath | 21 | 2000 | 1992-1998 | 33-39 S | 71-74 W | [[95](#_ENREF_95)] |
|  | 102 | Costa Rica | Golf of Dulce | 705 | Estuary | yes | Ecopath | 20 | 200 | 1990s | 8 N | 83 W | [[96](#_ENREF_96)] |
|  | 103 | Costa Rica | Gulf of Nicoya | 1,530 | Estuary | yes | Ecopath | 21 | 200 | 1990s | 9-10 N | 84-85 W | [[97](#_ENREF_97)] |
|  | 104 | Venezuela | Venzuela Shelf | 30,000 | Shelf | yes | Ecopath | 16 | 200 | 1980s | 10-11 N | 62-66 W | [[98](#_ENREF_98)] |
|  | 105 | Virgin Islands | Virgin Islands | 17 | Reef | no | Ecopath | 21 | 100 | 1960-1990s | 16-18 N | 60-67 S | [[99](#_ENREF_99)] |

* ENA – ecological network analysis, EwE = Ecopath and Ecosim

**References**

1. Stobberup KA, Ramos VDM, Coelho ML (2004) Ecopath model of the Cape Verde coastal ecosystem. In: Palomares ML, Pauly D, editors. West African marine ecosystems: models and fisheries impacts: Fisheries Center Research Reports 12(7). Vancouver, BC: UBC Fisheries Centre. pp. 39-56.

2. Mendy AN (2004) A trophic model of the Gambian continental shelf system in 1986. . In: Palomares MLD, Pauly D, editors. West African marine ecosystems: models and fisheries impacts Fisheries Centre Research Reports 12. Vancouver: UBC Fisheries Centre. pp. 81-94.

3. Pauly D (2002) Spatial modelling of trophic interactions and fisheries impacts in coastal ecosystems: A case study of Sakumo Lagoon, Ghana. In: McGlade JM, Cury P, Koranteng KA, Hardman-Mountford NJ, editors. The Gulf of Guinea Large Marine Ecosystem: Elsevier.

4. Diallo I, Cissé I, Bah A (2004) Modèle trophique du système côtier du plateau continental Guinéen. In: Palomares MLD, Pauly D, editors. West African marine ecosystems: models and fisheries impacts Fisheries Centre Research Reports 12. Vancouver: UBC Fisheries Centre. pp. 113-123.

5. Amorim PA, Duarte CM, Pires V, Geurra M, Morato T, et al. Improvements on the Guinea-Bissau Ecopath model with an exercise on simulating the effects of fishing; 2002 24-28 June 2002; Dakar, Senegal. EU Directorate-General of Research, International Scientific Cooperation. pp. 433-440.

6. Sidi TM, Guénette S (2004) Modèle trophique de la ZEE mauritanienne: comparaison de deux périodes (1987 et 1998). In: Palomares MLD, Pauly D, editors. West African marine ecosystems: models and fisheries impacts Fisheries Centre Research Reports 12. Vancouver: UBC Fisheries Centre. pp. 12-38.

7. Stanford R, Lunn K, Guénette S (2001) A preliminary ecosystem model for the Atlantic coast of Morocco in the mid-1980s. In: Guénette S Christensen V Pauly D (eds) Fish- eries impacts on North Atlantic ecosystems: models and analyses. Fish Cent Res Rep 9:314–344. In: Guénette S, Christensen V, Pauly D, editors. Fisheries impacts on North Atlantic ecosystems: mopdels and analyess Fisheries Centre Research Reports 9. Vancouver: UBC Fisheries Centre. pp. 314-344.

8. De Paula E Silva R, Sousa MI, Caramelo AM (1993) The Maputo Bay ecosystem (Mozambique). In: Christensen V, Pauly D, editors. Trophic models of aquatic ecosystems ICLARM Conference Proceedings 26. 1 ed. Manila, Philippines: International Center for Living Aquatic Resources Management. pp. 214-223.

9. Heymans JJ, Sumaila UR, Christensen V (2009) Policy options for the northern Benguela ecosystem using a multispecies, multifleet ecosystem model. Progress in Oceanography 83: 417-425.

10. Samb B, Mendy AN (2004) Dynamique du réseau trophique de l’écosystème Sénégambien en 1990. In: Palomares MLD, Pauly D, editors. West African marine ecosystems: models and fisheries impacts Fisheries Centre Research Reports 12. Vancouver: UBC Fisheries Centre. pp. 57-70.

11. Heymans JJ, Vakily JM (2002) Ecosystem structure and dynamics of the marine system of Sierra Leone for three time periods: 1964, 1978 and 1990. SIAP Tech. Doc. (SIAP/EP/DT/03) No. 3. In: Pauly D, Palomares MLD, Vakily JM, editors. Mass-balance trophic models of Northwest African marine ecosystems. Vancouver. pp. 109-120.

12. Vosloo MC (2004) A comparative assessment of the impact of recreational and subsistance fishing on selected Eastern Cape estuarine ecosystems using the ECOPATH modelling approach. Port Elizabeth: University of Port Elizabeth. 199 p.

13. Gribble NA (2003) GBR-prawn: modelling ecosystem impacts of changes in fisheries management of the commercial prawn (shrimp) trawl fishery in the far northern Great Barrier Reef. Fisheries Research 65: 493-506.

14. Mustafa MG. Trophic model of the coastal ecosystem in the waters of Bangladesh, Bay of Bengal. In: Silvestre G, Garces L, Stobutzki I, Ahmed M, Valmonte-Santos RA et al., editors; 2003. Worldfish Center Conference Proceedings 67. pp. 263-280.

15. Silvestre G, Selvanathan S, Salleh AHM (1993) Preliminary trophic model of the coastal fisheries resources of Brunei Darussalam, South China Sea. In: Christensen V, Pauly D, editors. Trophic models of aquatic ecosystems ICLARM Conference Proceedings 26. 1 ed. Manila, Philippines: International Center for Living Aquatic Resources Management. pp. 300-306.

16. Jiang H, Cheng H-Q, Xu H-G, Arreguín-Sánchez F, Zetina-Rejón MJ, et al. (2008) Trophic controls of jellyfish blooms and links with fisheries in the East China Sea. Ecological Modelling 212: 492-503.

17. Abdurahiman KP, Nayak TH, Zacharia PU, Mohamed KS (2010) Trophic organisation and predator-prey interactions among commercially exploited demersal finfishes in the coastal waters of the southeastern Arabian Sea. Estuarine, Coastal and Shelf Science 87: 601-610.

18. Vivekanandan E, Srinath M, Pillai VN, Immanuel S, Kurup KN. Trophic model of the coastal fisheries ecosystem of the Southwest Coast of India. In: Silvestre G, Garces L, Stobutzki I, Ahmed M, Valmonte-Santos RA et al., editors; 2003. Worldfish Center Conference Proceedings 67. pp. 281-298.

19. Garces LR, Man A, Ahmad AT, Mohamad-Norizam M, Silvestre GT. A trophic model of the coastal fisheries ecosystem off the West Coast of Sabah and Sarawak, Malaysia. In: Silvestre G, Garces L, Stobutzki I, Ahmed M, Valmonte-Santos RA et al., editors; 2003. Worldfish Center Conference Proceedings. pp. 333-352.

20. Bozec Y-M, Gascuel D, Kulbicki M (2004) Trophic model of lagoonal communities in a large open atoll (Uvea, Loyalty islands, New Caledonia). Aquatic Living Resources 17: 151-162.

21. Campos WL. An ecosystem model of San Pedro Bay, Leyte, Philippines: Initial parameter estimates. In: Silvestre G, Garces L, Stobutzki I, Ahmed M, Valmonte-Santos RA et al., editors; 2003. Worldfish Center Conference Proceedings 67. pp. 353-364.

22. Bundy A, Pauly D (2001) Selective harvesting by small-scale fisheries: ecosystem analysis of San Miguel Bay, Philippines. Fisheries Research 53: 263-281.

23. Lin H-J, Shao K-T, Hwang J-S, Lo W-T, Cheng I-J, et al. (2004) A trophic model for Kuosheng Bay in Northern Taiwan. Journal of Marine Science and Technology 12: 424-432.

24. Lin H-J, Shao K-T, Kuo S-R, Hsieh H-L, Wong S-L, et al. (1999) A trophic model of a sandy barrier lagoon at Chiku in Southwestern Taiwan. Estuarine, Coastal and Shelf Science 48: 575-588.

25. Christensen V, Walters CJ (2004) Trade-offs in Ecosystem-scale Optimization of Fisheries Management Policies. Bulletin of Marine Science 74: 549-562.

26. Vibunpant S, Khongchai N, Seng-eid J, Eiamsa-ard M, Supongpan M. Trophic model of the coastal fisheries ecosystem in the Gulf of Thailand. In: Silvestre G, Garces L, Stobutzki I, Ahmed M, Valmonte-Santos RA et al., editors; 2003. Worldfish Center Conference Proceedings 67. pp. 365-386.

27. Moreno T, Castro JJ (1998) Trophic structure of the Maspalomas lagoon (Gran Canaria, Canary Islands), a regenerated ecosystem of brackish water. Boletin do Museu Municipal do Funchal (História Natural) Sup. no. 5: 245-261.

28. Tomczak MT, Müller-Karulis B, Järv L, Kotta J, Martin G, et al. (2009) Analysis of trophic networks and carbon flows in south-eastern Baltic coastal ecosystems. Progress in Oceanography 81: 111-131.

29. Pinnegar JK, Polunin NVC (2004) Predicting indirect effects of fishing in Mediterranean rocky littoral communities using a dynamic simulation model. Ecological Modelling 172: 249-267.

30. Rybarczyk H, Elkaïm B, Ochs L, Loquet N (2003) Analysis of the trophic network of a macrotidal ecosystem: the Bay of Somme (Eastern Channel). Estuarine, Coastal and Shelf Science 58: 405-421.

31. Rybarczyk H, Elkaïm B (2003) An analysis of the trophic network of a microtidal estuary: the Seine Estuary (Eastern Channel, Normandy, France). Estuarine, Coastal and Shelf Science 58: 775-791.

32. Palomares ML, Reyes-Marchant P, Lair N, Zuinure M, Barnabé G, et al. (1993) A trophic model of a Mediterranean lagoon, Etang de Thau, France. In: Christensen V, Pauly D, editors. Trophic models of aquatic ecosystems ICLARM Conference Proceedings 26. 1 ed. Manila, Philippines: International Center for Living Aquatic Resources Management. pp. 224-229.

33. Lobry J, David V, Pasquaud S, Lepage M, Sautour B, et al. (2008) Diversity and stability of an estuarine trophic network. Marine Ecology Progress Series 358: 13-25.

34. Tsagarakis K, Coll M, Giannoulaki M, Somarakis S, Papaconstantinou C, et al. (2010) Food-web traits of the North Aegean Sea ecosystem (Eastern Mediterranean) and comparison with other Mediterranean ecosystems. Estuarine, Coastal and Shelf Science 88: 233-248.

35. Mendy AN (1998) Trophic modelling as a tool to evaluate and manage Iceland's multispecies fisheries. The United Nations University Fisheries Training Programme. 31 p.

36. Buchary EA (2001) Preliminary reconstruction of the Icelanding marine ecosystem in 1950 and some predictions with time series data. In: Guénette S, Christensen V, Pauly D, editors. Fisheries impacts on North Atlantic ecosystems: Models and analyses Fisheries Centre Researcg Reports 9(4). Vancouver BC: Fisheries Centre. pp. 198-206.

37. Brando VE, Ceccarelli R, Libralato S, Ravagnan G (2004) Assessment of environmental management effects in a shallow water basin using mass-balance models. Ecological Modelling 172: 213-232.

38. Carrer S, Opitz S (1999) Trophic network model of a shallow water area in the northern part of the Lagoon of Venice. Ecological Modelling 124: 193-219.

39. Libralato S, Pastres R, Pranovi F, Raicevich S, Granzotto A, et al. (2002) Comparison between the energy flow networks of two habitats in the Venice Lagoon. Marine Ecology 23: 228-236.

40. Libralato S, Tempesta M, Solidoro C, Spoto M (2006) Un modello di ecosistema applicato alla Riserva Naturale Marina di Miramare: Limiti, vantaggi e prospettive. Biol Mar Medit 13: 386-395.

41. Pranovi F, Libralato S, Raicevich S, Granzotto A, Pastres R, et al. (2003) Mechanical clam dredging in Venice lagoon: ecosystem effects evaluated with a trophic mass-balance model. Marine Biology 143: 393-403.

42. Coll M, Santojanni A, Palomera I, Arneri E (2009) Food-web changes in the Adriatic Sea over the last three decades. Marine Ecology Progress Series 381: 17-37.

43. Coll M, Santojanni A, Palomera I, Tudela S, Arneri E (2007) An ecological model of the Northern and Central Adriatic Sea: Analysis of ecosystem structure and fishing impacts. Journal of Marine Systems 67: 119-154.

44. Pedersen T, Nilsen M, Nilssen EM, Berg E, Reigstad M (2008) Trophic model of a lightly exploited cod-dominated ecosystem. Ecological Modelling 214: 95-111.

45. Coll M, Palomera I, Tudela S, Dowd M (2008) Food-web dynamics in the South Catalan Sea ecosystem (NW Mediterranean) for 1978-2003. Ecological Modelling 217: 95-116.

46. Coll M, Palomera I, Tudela S, Sardà F (2006) Trophic flows, ecosystem structure and fishing impacts in the South Catalan Sea, Northwestern Mediterranean. Journal of Marine Systems 59: 63-96.

47. Coll M, Bahamon N, Sardà F, Palomera I, Tudela S, et al. (2008) Improved trawl selectivity: effects on the ecosystem in the South Catalan Sea (NW Mediterranean). Marine Ecology Progress Series 355: 131-147.

48. Sánchez F, Olaso I (2004) Effects of fisheries on the Cantabrian Sea shelf ecosystem. Ecological Modelling 172: 151-174.

49. Anon. (2009) Baltic Sea 2020. "Best practices" for fisheries management. Baltic Nest Institute. Stockholm Resilience Centre. 96 p.

50. Hjerne O, Hansson S (2009) Documentation to MARE's fish Ecosim model. Stockholm: Department of Systems Ecology, Stockholm University. pp. 8.

51. Harvey CJ, Cox SP, Essington TE, Hansson S, Kitchell JF (2003) An ecosystem model of food web and fisheries interactions in the Baltic Sea. ICES J Mar Sci 60: 939-950.

52. Haggan N, Pitcher TJ (2005) Ecosystem simulation models of Scotland's West Coast and Sea Lochs. Vancouver: UBC Fisheries Centre. 1-67 p.

53. Sayer MD, Magill SH, Pitcher TJ, Morissette L, Ainsworth C (2005) Simulation-based investigations of fishery changes as affected by the scale and design of artificial habitats. Journal of Fish Biology 67: 218-243.

54. Araújo JN, Mackinson S, Stanford RJ, Hart PJB (2008) Exploring fisheries strategies for the western English Channel using and ecosystem model. Ecological Modelling 210: 465-477.

55. Araújo JN, Mackinson S, Stanford RJ, Sims DW, Southward AJ, et al. (2006) Modelling food web interactions, variation in plankton production, and fisheries in the western English Channel ecosystem. Marine Ecology Progress Series 309: 175-187.

56. Stanford R, Pitcher TJ (2004) Ecosystem simulations of the English Channel: Climate and trade-offs. Fisheries Centre Research Reports 12(3). Vancouver: Fisheries Centre. 12(3) 12(3). 103 p.

57. Mackinson S (2001) Representing trophic interactions in the North Sea in the 1880s, using the Ecopath mass-balance approach. In: Guénette S, Christensen V, Pauly D, editors. Fisheries impacts on North Atlantic ecosystems: Models and analyses Fisheries Centre Research Reports. Vancouver, BC: UBC FC. pp. 35-98.

58. Mackinson S, Daskalov G (2007) An ecosystem model of the North Sea for use in research supporting the ecosystem approach to fisheries management: description and parameterisation. Lowestoft: CEFAS. Cefas Science Series Technical Report 142 Cefas Science Series Technical Report 142. 200pp. p.

59. Heymans JJ, Howell KL, Ayers M, Burrows MT, Gordon JDM, et al. (2011) Do we have enough information to apply the ecosystem approach to management of deep-sea fisheries? An example from the West of Scotland. ICES Journal of Marine Science 68: 265-280.

60. Howell K, Heymans JJ, Gordon JDM, Ayers M, Jones E (2009) DEEPFISH Project: Applying an ecosystem approach to the sustainable management of deep-water fisheries. Part 1: Development of an Ecopath with Ecosim model. Oban: Scottish Association for Marine Science. 259a 259a. 116 p.

61. Mackinson S (1996) Strait of Georgia model. In: Pauly D, Christensen V, editors. Mass-balance models of North-eastern Pacific ecosystems Fisheries Centre Research Report 4(1). Vancouver BC: UBC. pp. 63-73.

62. Bundy A (2004) Mass balance models of the eastern Scotian Shelf before and after the cod collapse and other ecosystem changes. Dartmouth, NS: Department of Fisheries and Oceans. 2520 2520. 193 p.

63. Morissette L, Despatie S-P, Savenkoff C, Hammill MO, Bourdages H, et al. (2003) Data gathering and input parameters to construct ecosystem models for the northern Gulf of St. Lawrence (mid-1980s). Mont-Joli, Québec: DFO. 2497 2497. 102 p.

64. Savenkoff C, Castonguay M, Chabot D, Hammill MO, Bourdages H, et al. (2007) Changes in the northern Gulf of St. Lawrence ecosystem estimated by inverse modelling: Evidence of a fishery-induced regime shift? Estuarine, Coastal and Shelf Science 73: 711-724.

65. Rosado-Solórzano R, Guzmán del Próo SA (1998) Preliminary trophic structure model for Tampamachoco lagoon, Veracruz, Mexico. Ecological Modelling 109: 141-154.

66. Vega-Cendejas ME (2003) Trophic dynamics of a mangrove ecosystem in Celestun Lagoon, Yucatan Peninsula, Mexico. Fisheries Center Research Reports 11(6): 237-243.

67. Vega-Cendejas ME, Arreguín-Sánchez F (2001) Energy fluxes in a mangrove ecosystem from a coastal lagoon in Yucatan Peninsula, Mexico. Ecological Modelling 137: 119-133.

68. Zetina-Rejón M, Arreguín-Sánchez F, Chávez EA (2001) Using an ecosystem modelling approach to assess the management of a Mexican coastal lagoon system. CalCOFI Rep 42: 88-96.

69. Zetina-Rejón M, Arreguín-Sánchez F, Chávez EA (2003) Trophic structure and flows of energy in the Huizache-Caimanero lagoon complex on the Pacific coast of Mexico. Estuarine, Coastal and Shelf Science 57: 803-815.

70. Zetina-Rejón M, Arreguín-Sánchez F, Chávez EA (2004) Exploration of harvesting strategies for the management of a Mexican coastal lagoon fishery. Ecological Modelling 172: 361-372.

71. Vega-Cendejas ME, Arreguín-Sánchez F, Hernández M (1993) Trophic fluxes on the Campeche Bank, Mexico. In: Christensen V, Pauly D, editors. Trophic models of aquatic ecosystems ICLARM Conference Proceedings 26. 1 ed. Manila, Philippines: International Center for Living Aquatic Resources Management. pp. 206-212.

72. Abarca-Arenas LG, Valero-Pacheco E (1993) Towards a trophic model of Tamiahua, a coastal lagoon in Mexico. In: Christensen V, Pauly D, editors. Trophic models of aquatic ecosystems ICLARM Conference Proceedings 26. 1 ed. Manila, Philippines: International Center for Living Aquatic Resources Management. pp. 181-185.

73. Manickchand-Heileman S, Arreguín-Sánchez F, Lara-Domínguez A, Soto LA (1998) Energy flow and network analysis of Terminos Lagoon, SW Gulf of Mexico. Journal of Fish Biology 53: 179-197.

74. Rivera-Arriaga E, Lara-Domínguez AL, Villalobos-Zapata G, Yáñez-Arancibia A (2003) Trophodynamic ecology of two critical habitats (seagrasses and mangroves) in Términos Lagoon, southern Gulf of Mexico. Fisheries Center Research Reports 11(6): 245-254.

75. Morales-Zárate MV, Arreguín-Sánchez F, López-Martínez J, Lluch-Cota SE (2004) Ecosystem trophic structure and energy flux in the Northern Gulf of California, México. Ecological Modelling 174: 331-345.

76. Arreguín-Sánchez F, Calderón-Aguilera LE (2002) Evaluating harvesting strategies for fisheries in the Central Gulf of California ecosystem. In: Pitcher T, Cochrane K, editors. The Use of Ecosystem Models to Investigate Multispecies Management strategies for Capture Fisheries Fisheries Centre Research Reports 10(2). Vancouver: UBC Fisheries Centre. pp. 135-141.

77. Lercari D, Arreguín-Sánchez F (2009) An ecosystem modelling approach to deriving viable harvest strategies for multispecies management of the Northern Gulf of California. Aquatic Conservation: Marine and Freshwater Ecosystems 19: 384-397.

78. Manickchand-Heileman S, Soto LA, Escobar E (1998) A Preliminary Trophic Model of the Continental Shelf, South-western Gulf of Mexico. Estuarine, Coastal and Shelf Science 46: 885-899.

79. Arreguín-Sánchez F, Seijo JC, Valero-Pacheco E (1993) An application of Ecopath II to the North Continental Shelf Ecosystem of Yucatan, Mexico. In: Christensen V, Pauly D, editors. Trophic models of aquatic ecosystems ICLARM Conference Proceedings 26. 1 ed. Manila, Philippines: International Center for Living Aquatic Resources Management. pp. 269-278.

80. Browder JA (1993) A pilot model of the Gulf of Mexico Continental Shelf. In: Christensen V, Pauly D, editors. Trophic models of aquatic ecosystems ICLARM Conference Proceedings 26. 1 ed. Manila, Philippines: International Center for Living Aquatic Resources Management. pp. 279-284.

81. Arreguín-Sánchez F, del Monte-Luna P, Díaz-Uribe JG (2006) Trophic model for the ecosystem of La Paz Bay, on the Southern Baja California Peninsula, Mexico. INCOFISH models. Baja Californis Sur: Centro Interdisciplinario de Ciencias Marinas del IPN.

82. Arreguín-Sánchez F, Hernández-Herrera A, Ramírez-Rodríguez M, Pérez-España H (2004) Optimal management scenarios for the artisanal fisheries in the ecosystem of La Paz Bay, Baja California Sur, Mexico. Ecological Modelling 172: 373-382.

83. De La Cruz-Aguero G (1993) A preliminary model of Mandinga Lagoon, Veracruz, Mexico. In: Christensen V, Pauly D, editors. Trophic models of aquatic ecosystems ICLARM Conference Proceedings 26. 1 ed. Manila, Philippines: International Center for Living Aquatic Resources Management. pp. 193-196.

84. Arreguín-Sánchez F, Valero-Pacheco E, Chávez EA (1993) A trophic box model of the coastal fish communtities of the Southwestern Gulf of Mexico. In: Christensen V, Pauly D, editors. Trophic models of aquatic ecosystems ICLARM Conference Proceedings 26. 1 ed. Manila, Philippines: International Center for Living Aquatic Resources Management. pp. 197-205.

85. Dalsgaard J, Pauly D (1997) Preliminary mass-balance model of Prince William Sound, Alaska, for the pre-spill period, 1980-1989. Fisheries Centre Research Report 5: 1-33.

86. Okey TA, Wright BA (2004) Toward ecosystem-based extraction policies for Prince William Sound, Alaska: Integrating conflicting objectives and rebuilding pinnipeds. Bulletin of Marine Science 74: 727-747.

87. Heymans JJ (2005) Ecosystem model of the Western and Central Aleutian Islands in 1963, 1979 and 1991. In: Guénette S, Christensen V, editors. The Steller sea lion decline: models and data of the Northeast Pacific Fisheries Centre Research Reports 13(1). pp. 8-82.

88. Aydin KY, Lapko VV, Radchenko VI, Livingston PA (2002) Comparison of the Eastern Bering and Western Bering Sea shelf and slope ecosystems Through the use of mass-balance food web models. NMFS AFSC 130 NMFS AFSC 130. 92 p.

89. Olivieri RA, Cohen A, Chavez FP (1993) An ecosystem model of Monterey Bay, California. In: Christensen V, Pauly D, editors. Trophic models of aquatic ecosystems ICLARM Conference Proceedings 26. 1 ed. Manila, Philippines: International Center for Living Aquatic Resources Management. pp. 315-322.

90. Venier JM, Pauly D. Trophic dynamics of a Florida Keys coral reef ecosystem; 1997; Panama City. Smithsonian Tropica Research Institute, Balboa, Panama. pp. 915-920.

91. Okey TA, Vargo GA, Mackinson S, Vasconcellos M, Mahmoudi B, et al. (2004) Simulating community effects of sea floor shading by plankton blooms over the West Florida Shelf. Ecological Modelling 172: 339-359.

92. Vasconcellos M, Gasalla MA (2001) Fisheries catches and the carrying capacity of marine ecosystems in southern Brazil. Fisheries Research 50: 279-295.

93. Gasalla MA, Rossi-Wongtschowski CLDB (2004) Contribution of ecosystem analysis to investigating the effects of changes in fishing strategies in the South Brazil Bight coastal ecosystem. Ecological Modelling 172: 283-306.

94. Wolff M (1994) A trophic model for Tongoy Bay - a system exposed to suspended scallop culture (Northern Chile). Journal of Experimental Marine Biology and Ecology 182: 149-168.

95. Neira S, Arancibia H, Cubillos L (2004) Comparative analysis of trophic structure of commercial fishery species off Central Chile in 1992 and 1998. Ecological Modelling 172: 233-248.

96. Wolff M, Hartmann HJ, Koch V (1996) A pilot trophic model for Golfo Dulce, a fjord-like tropical embayment, Costa Rica. Revista de Biología Tropical 44: Supl. 3: 215-231.

97. Wolff M, Koch V, Chavarría-Chaves JB, Vargas-Zamora JA (1998) A trophic flow model of the Golfo de Nicoya, Costa Rica. Revista de Biología Tropical 46: Supl. 6: 63-79.

98. Mendoza JJ (1993) A preliminary biomass budget for the Northeastern Venezuela shelf ecosystem. In: Christensen V, Pauly D, editors. Trophic models of aquatic ecosystems ICLARM Conference Proceedings 26. 1 ed. Manila, Philippines: International Center for Living Aquatic Resources Management. pp. 285-297.

99. Opitz S (1993) A quantitative model of the trophic interactions in a Caribbean coral reef ecosystem. In: Christensen V, Pauly D, editors. Trophic models of aquatic ecosystems ICLARM Conference Proceedings 26. 1 ed. Manila, Philippines: International Center for Living Aquatic Resources Management. pp. 259-267.
