# Supplementary material for: Global Patterns in Ecological Indicators of Marine Food Webs: A Modelling Approach
Source: PLoS One. 2014 Apr 24;9(4):e95845. doi: 10.1371/journal.pone.0095845 (PMC3998982; doi:10.1371/journal.pone.0095845)
Supplement: File S1 — Description of Ecopath with Ecosim. (DOCX) [file pone.0095845.s002.docx]

### Supporting Information S2: Ecopath with Ecosim

The modelled food web is represented by nodes or functional groups (*i*), which can be composed of species, groups of species with ecological similarities or ontogenetic fractions of a species. Ecopath uses two equations to parameterize models: one for the energy balance of each group and one to describe the production [[1](#_ENREF_1)]. The production of each group is estimated using the equation [[1](#_ENREF_1)]:

*Production = catch + predation mortality + biomass accumulation + net migration*

*+ other mortality* (1)

or, more formally:

$P_{i}=Y_{i}+B_{i}\cdot M2_{i}+E_{i}+BA_{i}+P_{i}\cdot(1-EE_{i})$ (2)

where *Pi* is the total production of group *i*, *Yi* is the total fishery catch rate of *i*, *M2i* is the instantaneous predation rate for group *i*, *Ei* the net migration rate (emigration - immigration), *BAi* is the biomass accumulation rate for *i*, and *Pi·(1-EEi)* is the ‘other mortality’ rate for *i* [[1](#_ENREF_1)]. Equation (2) can be re-written as:

$B_{i}\cdot(P/B)_{i}\cdot EE_{i}-\sum_{j=1}^{n} B_{j}\cdot(Q/B)_{j}\cdot DC_{ji}-Y_{i}-E_{i}-BA_{i}=0$ (3)

where *P/Bi* is the production/biomass ratio for *i* and under most conditions corresponds to the total mortality rate, Z, commonly estimated as part of fishery stock assessment. *EEi* is the ecotrophic efficiency of group *i*, describing the proportion of the production that is utilised in the system, Q*/Bj* is the consumption/biomass ratio of the predator *j* and *DCji* is the fraction of prey *i* in the average diet of predator *j* [[1](#_ENREF_1)].

The energy balance within each group is ensured when consumption by a group i (Qi)

Qi = Pi + Ri + UAi (4)

Where Pi is total production, Ri is respiration and UAi is unassimilated food of group i.

The model is built using nutrient or energy related currencies per unit of surface. Frequently biomass is expressed as t·km^-2^ and flows are expressed as t·km^-2^·yr^-1^.

Ecopath requires three of the following four data for each group of the model:

- Biomass (B, t·km^-2^) for the year under consideration
- Production/Biomass ratio (P/B, year^-1^);
- Consumption/Biomass ratio (Q/B, year^-1^);
- Ecotrophic Efficiency (proportion). This parameter indicates the unexplained mortality for each group.

In addition for each group the diet composition is required as a contribution (in mass) of the prey items in the diet of the group and for each fishery the group specific landings (t·km^-2^·year^-1^) and discards (t·km^-2^·year^-1^) are required.

**Reference:**

1. Christensen V, Walters C, Pauly D (2005) Ecopath with Ecosim: A User's guide. Vancouver, BC: Fisheries Centre, University of British Columbia. 154pp. p.
